# Activated Macrophages Promote TNF-α-Associated Tumor Cell Necroptosis in Pituitary Apoplexy Through the PIEZO1–NFATC2/REL Axis

**DOI:** 10.3390/ijms27125635

**Published:** 2026-06-22

**Authors:** Xingbo Li, Luowen Zhou, Zhuowei Lei, Sihan Li, Quanji Wang, Haochen Zhao, Linpeng Xu, Juan Chen, Xueyan Wan, Yimin Huang, Ting Lei

**Affiliations:** 1 Sino-German Neuro-Oncology Molecular Laboratory, Department of Neurosurgery, Tongji Hospital of Tongji Medical College of Huazhong University of Science and Technology, Wuhan 430030, China; lxbhusttj@tjh.tjmu.edu.cn (X.L.); d202282086@hust.edu.cn (L.Z.); tjlzw2018@tjh.tjmu.edu.cn (Z.L.); lisihan@hust.edu.cn (S.L.); quanjiwang@hust.edu.cn (Q.W.); u201810325@hust.edu.cn (H.Z.); m202276296@hust.edu.cn (L.X.); 2 Hubei Key Laboratory of Neural Injury and Functional Reconstruction, Huazhong University of Science and Technology, Wuhan 430030, China; jchen@tjh.tjmu.edu.cn (J.C.); xywan@tjh.tjmu.edu.cn (X.W.); 3 Department of Orthopedics, Tongji Hospital of Tongji Medical College of Huazhong University of Science and Technology, Wuhan 430030, China

**Keywords:** pituitary apoplexy, pituitary neuroendocrine tumor, macrophages, mechanotransduction, PIEZO1, NFATC2/REL, TNF-α, necroptosis

## Abstract

Pituitary apoplexy is an uncommon but clinically urgent complication that often involves intrasellar hemorrhage and tissue necrosis. The mechanisms linking acute tissue injury to the inflammatory tumor microenvironment remain incompletely defined. Here, we characterized the apoplexy-associated microenvironment and examined whether macrophage mechanosensitive signaling contributes to inflammatory amplification and tissue damage in pituitary neuroendocrine tumors (PitNETs). We combined single-cell RNA sequencing (scRNA-seq), histological validation, clinical stratification, and in vitro functional assays using apoplectic and non-apoplectic human PitNET specimens. Macrophage state transitions, intercellular communication, and transcriptional regulatory programs were analyzed, followed by an experimental assessment of the PIEZO1–Ca^2+^ axis and macrophage-conditioned medium-induced tumor cell death. Histological validation confirmed macrophage accumulation in apoplectic PitNETs, including a 1.67-fold increase in IBA-1-positive cells (*p* < 0.001). CellChat-inferred interaction metrics increased descriptively in apoplectic samples. Apoplectic tissues showed higher TNF-α expression (3.00-fold; *p* < 0.0001) and higher PIEZO1 fluorescence in IBA-1-positive regions (1.39-fold; *p* = 0.001). Yoda1 increased Calcium 520 fluorescence in macrophages (1.72-fold; *p* = 0.002), whereas *Piezo1* knockdown reduced the Yoda1-associated response (*p* = 0.003). Conditioned medium from activated macrophages increased total Annexin V/PI-positive death in AtT-20 cells (0.53 ± 0.53% to 32.48 ± 1.14%; *p* < 0.001) and GH3 cells (0.82 ± 0.50% to 30.92 ± 1.11%; *p* < 0.001); *Piezo1* knockdown or TNF-α neutralization attenuated this effect. Clinically, pathological necrosis was associated with higher symptom frequencies and a greater adjusted likelihood of two or more clinical symptoms. Together, these findings indicate that PIEZO1-related macrophage signaling may participate in TNF-α-associated tumor cell necroptosis in pituitary apoplexy. Pathological necrosis was linked to greater acute symptom burden and perioperative hormonal abnormalities, suggesting that it may identify a clinically severe apoplexy subtype.

## 1. Introduction

Pituitary neuroendocrine tumors (PitNETs) are common sellar neoplasms and represent a major category of neuroendocrine tumors in clinical practice [1,2]. Pituitary apoplexy is among their most severe acute complications and can present with sudden headache, visual deterioration, ophthalmoplegia, and variable hypopituitarism; in severe cases, it may be life-threatening [2,3]. Pathologically, apoplexy encompasses hemorrhage, infarction, hemorrhage after infarction, and infarction after hemorrhage. Necrotic lesions usually follow two patterns: hemorrhagic/coagulative necrosis and ischemic necrosis [4,5]. Early lesions are dominated by hemorrhage or isolated infarction [6], whereas later lesions may contain calcification, fibrosis, or granulation tissue [7,8]. On hematoxylin and eosin (H&E) staining, apoplectic tumors often show extensive necrosis, ghost-like cell outlines, and the loss of nuclear detail; necrotic foci may also contain fibrous deposition and weak or absent immunoreactivity. The biological events that connect these histological changes to the acute clinical syndrome remain poorly understood.

Several mechanisms have been proposed for pituitary apoplexy, including elevated intratumoral pressure, inadequate local blood supply, increased metabolic demand, hypoxia, and abnormal angiogenesis [9]. Our previous studies found that inflammatory and vascular remodeling mediators, including TNF-α, MMPs, and VEGF, were upregulated in apoplectic pituitary tumor tissues [10,11]. Such factors may influence vascular instability and extracellular matrix remodeling during apoplexy [12]. Whether they act as downstream markers of tissue injury or as active contributors to disease progression, however, remains unresolved.

The tumor microenvironment (TME) provides a plausible framework for exploring the cellular sources of the above mediators. PitNETs are not immunologically inert tumors; single-cell RNA sequencing (scRNA-seq) and spatial transcriptomic studies have progressively shown that their microenvironment comprises immune cells, vascular-associated cells, and diverse stromal components [13,14,15]. Among immune cell subsets, tumor-associated macrophages (TAMs) represent a prominent infiltrating population in PitNETs and have been associated with tumor progression, invasive behavior, and peritumoral microenvironmental remodeling [16,17,18]. Notably, inflammatory, matrix remodeling, and pro-angiogenic mediators, including TNF-α, MMPs, and VEGF, can be produced by activated macrophages or TAMs [19,20]. These observations suggest that TAMs may contribute to the inflammatory signaling milieu in apoplectic tumor tissues.

Macrophage activation is shaped by multiple microenvironmental cues, including inflammatory mediators, metabolic alterations, cell–cell interactions, and extracellular matrix remodeling [19,21]. Among these cues, biomechanical signals within the microenvironment have recently been recognized as regulators of macrophage activation states [22]. The pituitary gland is located within the relatively confined sellar region, and tumor growth, local hemorrhage, and changes in tissue tension may collectively indicate the presence of an altered mechanical microenvironment in this anatomical compartment [23]. In addition, previous studies have reported that intrasellar pressure is associated with tumor volume and invasive growth features [24]. In the context of pituitary apoplexy, these findings raise the possibility that local mechanical abnormalities may act as microenvironmental signals involved in immune cell activation, inflammatory amplification, and tissue injury. The PIEZO family comprises evolutionarily conserved mechanosensitive ion channels that sense pressure, stretch, and matrix stiffness and convert extracellular physical stimuli into intracellular signaling events [25,26]. In macrophages, PIEZO channel activation has been reported to induce Ca^2+^ influx, NF-κB nuclear translocation, and the transcriptional activation of pro-inflammatory genes, including TNF-α [22,27]. Accordingly, whether local mechanical abnormalities may contribute to inflammatory amplification and tissue injury in pituitary apoplexy through PIEZO1-mediated macrophage mechanotransduction represents a key question addressed in the present study.

The restricted anatomy of the sellar region provides a plausible mechanical context for apoplexy. Intratumoral pressure increases with pituitary adenoma size, and an abnormal mechanical microenvironment has been proposed as a potential trigger for acute tumor injury. PIEZO1, a mechanosensitive ion channel, detects pressure, stretch, and matrix stiffness and converts these extracellular cues into intracellular signaling events, including inflammation-related responses [18,28]. Our prior observations indicated increased PIEZO1 expression in apoplectic pituitary tumor tissues. This raised the possibility that local mechanical stress may contribute to inflammatory mediator expression through PIEZO1-dependent signaling.

Building on our earlier findings of increased TNF-α, MMPs, and other inflammatory mediators [10,11], together with elevated PIEZO1 expression in apoplectic tissues, we investigated whether PIEZO1 signaling is linked to TNF-α-centered macrophage inflammation in pituitary apoplexy. This study was designed to connect single-cell microenvironmental changes, mechanosensitive macrophage activation, tumor cell necroptosis, and the clinical phenotype of necrotic apoplexy.

## 2. Results

### 2.1. Apoplexy-Associated Immune Microenvironmental Remodeling in PitNETs

**Figure 1:**
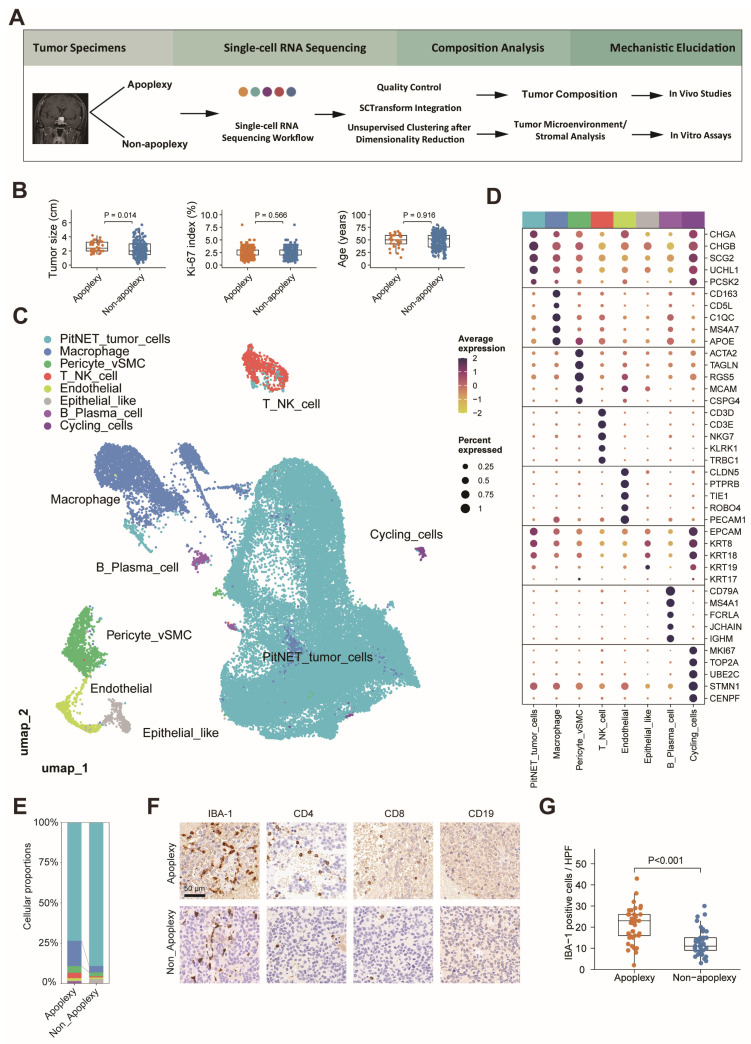
Macrophage-enriched immune remodeling in apoplectic PitNETs. (**A**) Study workflow integrating clinical specimens, single-cell profiling, tissue validation, and functional assays. (**B**) Tumor size, Ki-67 index, and age comparisons between groups, with *p* values indicated. (**C**) UMAP of annotated major cell populations. (**D**) Marker gene dot plot supporting cell type annotation. (**E**) Relative cell type proportions in apoplectic and non-apoplectic samples. (**F**) IHC staining for IBA-1, CD4, CD8, and CD19 in apoplectic and non-apoplectic PitNET tissues. (**G**) Quantification of IBA-1-positive cells per high-power field.

We profiled apoplectic and non-apoplectic PitNET specimens using scRNA-seq and histological validation (Figure 1A). Tumor size was larger in the apoplexy group than in the non-apoplexy group (2.61 ± 0.74 vs. 2.27 ± 1.00 cm; *p* = 0.014), whereas the Ki-67 index and age did not differ significantly between groups (Figure 1B). Integrated UMAP analysis resolved the major cell populations, including PitNET tumor cells, macrophages, pericytes/vascular smooth muscle cells (vSMCs), T and natural killer (NK) cells, endothelial cells, epithelial-like cells, B and plasma cells, and proliferating/cycling cells; canonical marker genes supported these annotations (Figure 1C,D). Sample-level UMAPs, cell type proportion heatmaps, enrichment heatmaps, and marker gene heatmaps further supported the annotation and sample-level composition (Figure S1A–D). Relative composition analysis showed that PitNET tumor cells formed the dominant compartment and that macrophages represented the major immune population enriched in apoplectic specimens (Figure 1E and Figure S1E). Histological validation confirmed higher IBA-1-positive cell density in apoplectic tissues (1.67-fold; *p* < 0.001), along with increases in CD4-positive cells (2.00-fold; *p* < 0.001), CD8-positive cells (4.21-fold; *p* < 0.001), and CD19-positive cells (*p* < 0.001) (Figure 1F,G and Figure S1F–H). Thus, apoplectic PitNETs exhibited immune microenvironmental remodeling, with macrophage accumulation as a prominent feature.

### 2.2. Inflammatory Activation of Apoplexy-Associated Macrophages

**Figure 2:**
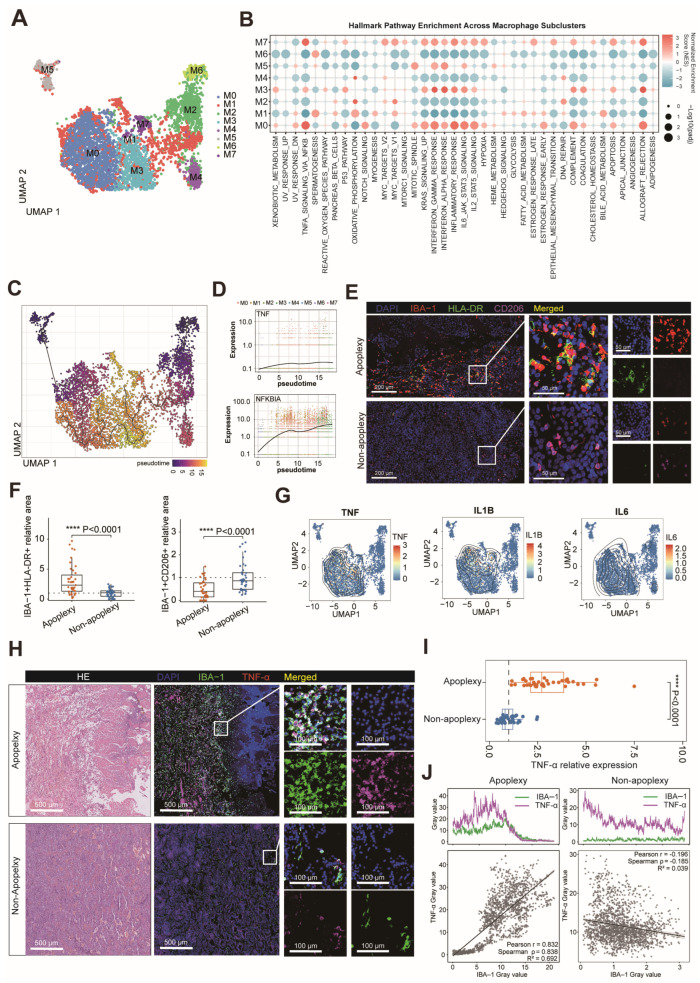
Inflammatory activation and TNF-α enrichment in apoplexy-associated macrophages. (**A**) UMAP of macrophage transcriptomic subclusters M0–M7. (**B**) Hallmark pathway enrichment across macrophage subclusters. (**C**) Macrophage pseudotime trajectory. (**D**) *TNF* and *NFKBIA* expression dynamics along pseudotime. (**E**) Multiplex IF staining for DAPI, IBA-1, HLA-DR, and CD206. (**F**) Quantification of IBA-1/HLA-DR and IBA-1/CD206 relative areas. (**G**) Feature plots of *TNF*, *IL1B*, and *IL6* in macrophages. (**H**) H&E and IBA-1/TNF-α multiplex IF staining in apoplectic and non-apoplectic tissues. (**I**) Relative TNF-α expression. (**J**) Spatial intensity profiles and correlation analysis of IBA-1 and TNF-α signals. Statistical significance was defined as **** *p* < 0.0001.

Macrophages were reclustered into eight unsupervised transcriptional subclusters (M0–M7) (Figure 2A). These labels do not denote predefined classical M0/M1/M2 macrophage states; instead, cluster interpretation was based on marker genes, pathway enrichment, pseudotime/regulon analysis, and multiplex IF validation. Therefore, M0–M7 are referred to as transcriptomic subclusters, whereas M1-like and M2-like are used only for HLA-DR- and CD206-associated IF phenotypes, respectively. Macrophage subcluster marker heatmaps and dot plots supported the data-driven annotation, and the apoplexy and non-apoplexy groups showed distinct macrophage subcluster distributions and proportions (Figure S2A–D). Hallmark enrichment analysis linked apoplexy-enriched macrophage subsets to inflammatory pathways, including TNF-α signaling via NF-κB, inflammatory response, and IL6-JAK-STAT3 signaling. Along pseudotime, *TNF* and *NFKBIA* increased progressively (Figure 2B–D). In the main tissue panel, the IBA-1/HLA-DR-positive M1-like area increased (3.12-fold; *p* < 0.0001), whereas the IBA-1/CD206-positive M2-like area decreased (0.49-fold; *p* < 0.0001) (Figure 2E,F and Figure S2E). Feature plots detected *TNF*, *IL1B*, and *IL6* expression across macrophage subsets (Figure 2G). H&E staining combined with IBA-1/TNF-α multiplex IF further showed higher TNF-α relative expression in apoplectic tissues (3.00-fold; *p* < 0.0001) and stronger spatial alignment between IBA-1 and TNF-α signals in apoplexy than in non-apoplexy (Figure 2H–J).

### 2.3. PIEZO1-Associated Inflammatory Regulation in Macrophages

**Figure 3:**
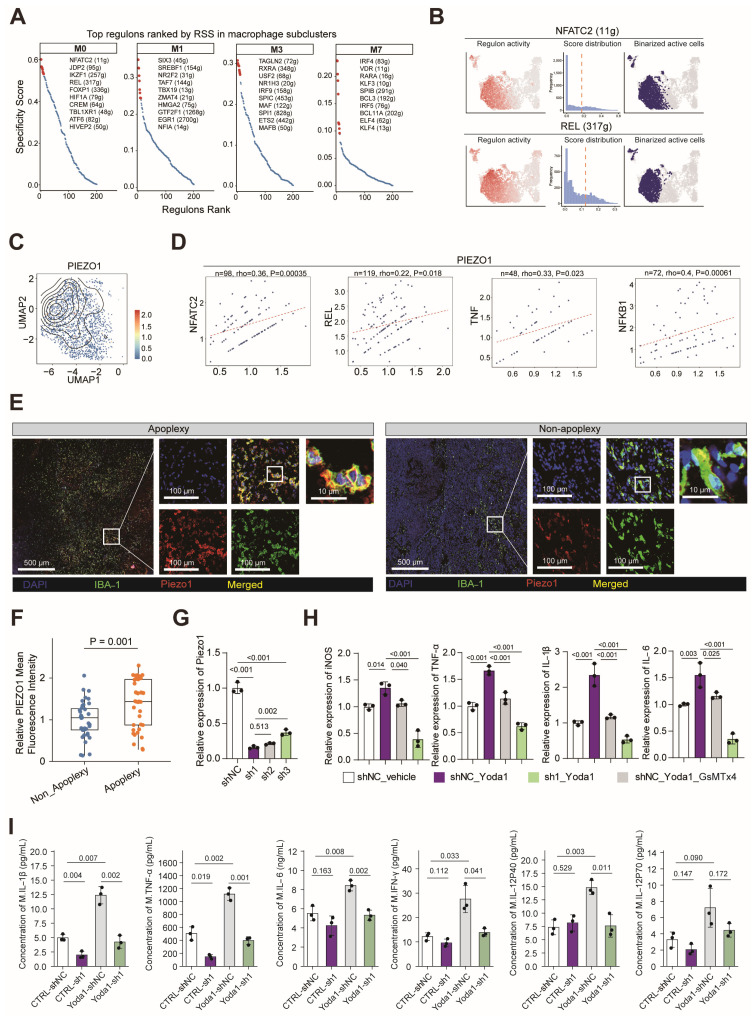
PIEZO1 links inflammatory macrophage regulons with cytokine output. (**A**) Regulon specificity scores for representative macrophage subclusters M0, M1, M3, and M7. (**B**) *NFATC2* and *REL* regulon activity, score distribution, and active cell maps. (**C**) *PIEZO1* expression in macrophages. (**D**) Correlations between *PIEZO1* and *NFATC2*, *REL*, *TNF*, or *NFKB1*, with sample sizes, Spearman ρ values, and *p* values indicated. (**E**) Multiplex IF staining for DAPI, IBA-1, and PIEZO1 in apoplectic and non-apoplectic tissues. (**F**) Quantification of PIEZO1 fluorescence intensity. (**G**) RT-qPCR validation of *Piezo1* knockdown in RAW264.7 cells. (**H**) RT-qPCR analysis of *Nos2*, *Tnf*, *Il1b*, and *Il6* mRNA expression after Yoda1, *Piezo1* knockdown, or GsMTx4 treatment. (**I**) Cytokine measurements in macrophage culture supernatants.

Macrophage regulon analysis identified *NFATC2* as a prominent regulon in selected macrophage subclusters, whereas NF-κB-related regulators also ranked prominently (Figure 3A and Figure S3A). *REL* regulon activity occupied adjacent macrophage regions, and cells with high *NFATC2* or *REL* activity showed neighboring or partially overlapping distributions (Figure 3B and Figure S3B,C). Because NFAT nuclear translocation is controlled by the Ca^2+^/calmodulin–calcineurin axis [29], and Ca^2+^ signaling can modulate NF-κB/Rel activity through its amplitude, duration, and oscillatory pattern [30], we examined calcium-channel-related signaling. *PIEZO1* was locally expressed in the macrophage UMAP and showed positive correlations with key inflammatory regulons and mediators, including *NFATC2* (*p* < 0.001), *REL* (*p* = 0.018), *TNF* (*p* = 0.023), and *NFKB1* (*p* < 0.001), whereas *PIEZO2*-related analyses, including positive correlations with *IL1B* and *RELA*, are provided in the Supplementary Data (Figure 3C,D and Figure S3D–F). In tissue multiplex IF, PIEZO1 mean fluorescence intensity in IBA-1-positive regions was higher in apoplectic than non-apoplectic specimens (1.39-fold; *p* = 0.001) (Figure 3E,F). We then silenced *Piezo1* in RAW264.7 cells (Figure 3G). Yoda1 increased *Nos2*, *Tnf*, *Il1b*, and *Il6* mRNA expression, whereas *Piezo1* knockdown or GsMTx4 treatment reduced these inflammatory responses (Figure 3H). Cytokine profiling showed a similar pattern: Yoda1 increased the principal inflammatory cytokines relative to Ctrl_NC (IL-1β, *p* = 0.007; TNF-α, *p* = 0.002; IL-6, *p* = 0.008), whereas *Piezo1* knockdown reduced the Yoda1-associated cytokine signals (Yoda1_NC vs. Yoda1_Sh: IL-1β, *p* = 0.002; TNF-α, *p* = 0.001; IL-6, *p* = 0.002) (Figure 3I and Figure S3G).

### 2.4. Activation of PIEZO1-Ca^2+^-NFATC2/REL Axis

**Figure 4:**
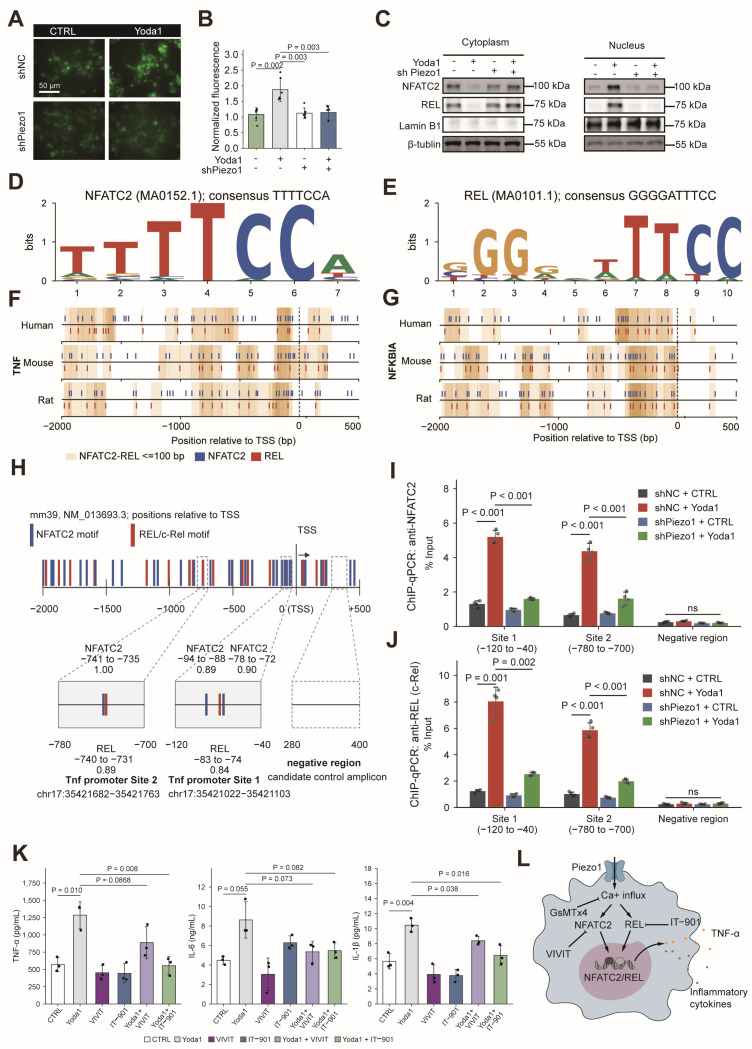
PIEZO1 activation engages Ca^2+^-NFATC2/REL signaling and inflammatory promoter occupancy. (**A**) Calcium 520 fluorescence in shNC and sh*Piezo1* macrophages with or without Yoda1 stimulation. (**B**) Quantification of Calcium 520 fluorescence intensity, with planned comparison *p* values indicated. (**C**) Western blot analysis of NFATC2 and REL in cytoplasmic and nuclear fractions. (**D**,**E**) NFATC2 and REL motif logos. (**F**,**G**) NFATC2/REL motif distribution in *TNF* and *NFKBIA* promoter regions across human, mouse, and rat sequences. (**H**) Mouse *Tnf* promoter design for ChIP-qPCR. (**I**,**J**) NFATC2 and REL/c-Rel ChIP-qPCR enrichment at *Tnf* promoter Site 1, Site 2, and negative control region. (**K**) TNF-α, IL-6, and IL-1β secretion after Yoda1, VIVIT, or IT-901 treatment. (**L**) Proposed PIEZO1-Ca^2+^-NFATC2/REL inflammatory signaling model.

Yoda1 increased normalized Calcium 520 fluorescence in shNC macrophages (*p* = 0.002), whereas *Piezo1* knockdown reduced the Yoda1-associated response (*p* = 0.003 for the comparisons indicated in Figure 4B). Nuclear/cytoplasmic fractionation followed by Western blotting showed increased nuclear NFATC2 and REL after Yoda1 treatment, whereas *Piezo1* knockdown limited their nuclear enrichment (Figure 4C).

Motif analysis identified consensus NFATC2 and REL sequences positioned in proximity within promoter regions of inflammatory genes, including *TNF, NFKBIA, IL1B, IL6, NFKB2*, and *NFKBID* (Figure 4D–G and Figure S4A–F). ChIP-qPCR was then designed around Site 1, Site 2, and a negative region in the mouse *Tnf* promoter. In WT macrophages, Yoda1 increased NFATC2 enrichment at *Tnf* Site 1 and Site 2 (both *p* < 0.001), and *Piezo1* knockdown reduced Yoda1-associated NFATC2 enrichment at both sites (both *p* < 0.001), whereas the negative region remained non-significant (Figure 4I). REL/c-Rel showed the same pattern: at Site 1, Yoda1 increased enrichment (*p* = 0.001), and *Piezo1* knockdown reduced the Yoda1-associated signal (*p* = 0.002); at Site 2, both corresponding comparisons were *p* < 0.001, while the negative region remained non-significant (Figure 4J). Similar changes were detected at *Il6* and *Il1b* promoter-associated sites (Figure S4G,H). In inhibitor assays, Yoda1 increased TNF-α secretion (569.23 ± 103.26 to 1286.40 ± 190.66 pg/mL; *p* = 0.010) and IL-1β secretion (5.67 ± 1.02 to 10.46 ± 0.91 pg/mL; *p* = 0.004). IT-901 significantly reduced Yoda1-associated TNF-α and IL-1β secretion (Yoda1 vs. Yoda1 + IT-901: TNF-α, *p* = 0.008; IL-1β, *p* = 0.016), whereas VIVIT reduced IL-1β (Yoda1 vs. Yoda1 + VIVIT: *p* = 0.038). The remaining inhibitor comparisons did not reach *p* < 0.05 and are described as trends only (Figure 4K). Together, these in vitro data link Yoda1-mediated PIEZO1-related calcium signals with NFATC2/REL nuclear localization, promoter occupancy, and inflammatory cytokine secretion (Figure 4L).

### 2.5. Apoplexy-Associated Cell–Cell Communication and Cell Death Signaling

**Figure 5:**
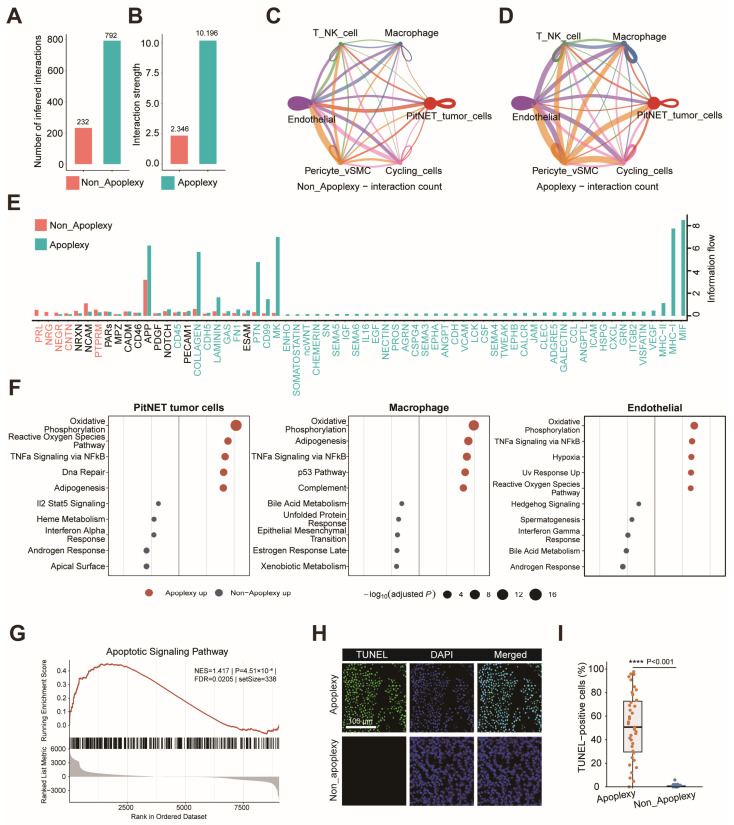
Strengthened cell–cell communication and cell death signatures in apoplectic PitNETs. (**A**,**B**) CellChat-inferred interaction number and overall interaction strength in non-apoplectic and apoplectic samples. (**C**,**D**) Inferred interaction count networks among major cell populations. (**E**) Relative information flow across ligand–receptor pathways. (**F**) Hallmark pathway enrichment in group-upregulated genes in PitNET tumor cells, macrophages, and endothelial cells. (**G**) GSEA of apoptotic signaling pathway in PitNET tumor cells, with NES, nominal *p* value, FDR, and gene set size indicated. The red line indicates the running enrichment score, the black barcode lines indicate gene-set positions, and the grey area indicates the ranked-list metric. (**H**,**I**) TUNEL staining and quantification in apoplectic and non-apoplectic tissues.

Grouped UMAP and differential expression analyses revealed stress-, inflammation-, and vascular-related transcriptional changes across multiple cell populations in apoplectic specimens (Figure S5A,B). CellChat analysis indicated descriptively higher inferred interaction numbers and overall interaction strength in the apoplexy group: the inferred interaction number increased from 232 to 792 (3.41-fold), and the overall interaction strength increased from 2.346 to 10.196 (4.35-fold), accompanied by a denser communication network among major cell populations (Figure 5A–D and Figure S5C,D). Outgoing/incoming signaling patterns suggested that multiple apoplexy-associated cell populations acted as both senders and receivers, and relative information flow highlighted enhanced immune, adhesion, extracellular matrix, and vascular programs, including MIF and MHC-I/II signaling (Figure 5E and Figure S5E). Hallmark enrichment highlighted inflammation-, stress-, and cell-death-related pathways, including TNF-α signaling via NF-κB and the p53 pathway (Figure 5F and Figure S5F). In PitNET tumor cells, GSEA enriched the apoptotic signaling pathway (NES = 1.417, *p* = 4.51 × 10^−4^, FDR = 0.0205; Figure 5G). Consistent with these transcriptomic signatures, TUNEL positivity was higher in apoplectic than non-apoplectic tissues (78.10-fold; *p* < 0.001) (Figure 5H,I). These findings indicate that apoplectic PitNETs harbor strengthened intercellular communication and increased cell death signaling.

### 2.6. Macrophage-Conditioned Medium Induces TNF-α-Associated Necroptotic Tumor Cell Death

**Figure 6:**
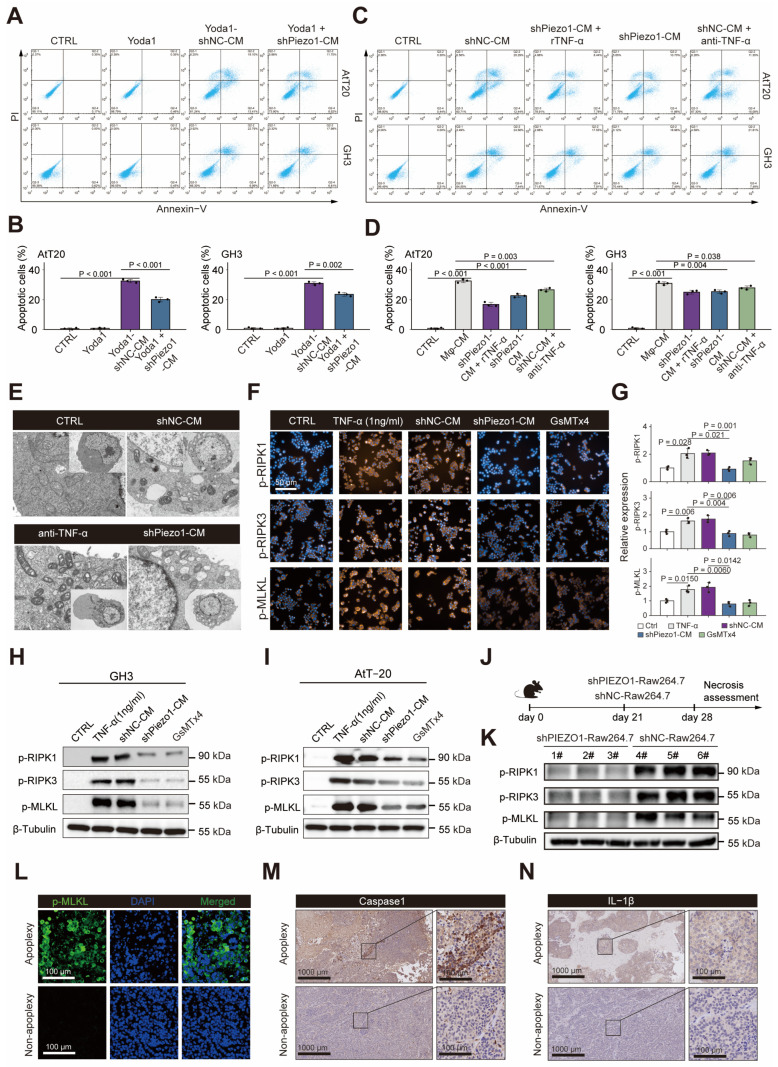
Macrophage-derived TNF-α promotes necroptosis-associated tumor cell death. (**A**,**B**) Annexin V/PI cell death analysis and quantification of AtT-20 and GH3 cells after control treatment, Yoda1 treatment, conditioned medium from Yoda1-activated shNC macrophages (Yoda1-shNC-CM), or conditioned medium from Yoda1-activated sh*Piezo1* macrophages (Yoda1-sh*Piezo1*-CM). (**C**,**D**) Annexin V/PI cell death analysis and quantification after shNC-CM, sh*Piezo1*-CM plus recombinant TNF-α (rTNF-α), sh*Piezo1*-CM, or shNC-CM plus anti-TNF-α treatment. (**E**) TEM assessment of tumor cell ultrastructural injury after control, shNC-CM, anti-TNF-α, or sh*Piezo1*-CM treatment. (**F**,**G**) IF staining and quantification of p-RIPK1, p-RIPK3, and p-MLKL after control, TNF-α, shNC-CM, sh*Piezo1*-CM, or GsMTx4 treatment. (**H**,**I**) Western blot analysis of RIPK1/RIPK3/MLKL pathway readouts in GH3 and AtT-20 cells, respectively. (**J**) In vivo experimental design. (**K**) Western blot analysis of p-RIPK1, p-RIPK3, and p-MLKL in tumor tissues from sh*Piezo1*-RAW264.7 and shNC-RAW264.7 groups. (**L**) Clinical tissue IF staining for p-MLKL. (**M**,**N**) Clinical tissue IHC staining for Caspase-1 and IL-1β, respectively.

Conditioned medium from Yoda1-activated shNC macrophages increased Annexin V/PI-positive death in AtT-20 cells (0.53 ± 0.53% to 32.48 ± 1.14%, *p* < 0.001) and GH3 cells (0.82 ± 0.50% to 30.92 ± 1.11%, *p* < 0.001), whereas macrophage *Piezo1* knockdown attenuated this effect (AtT-20: 20.06 ± 1.55%, *p* < 0.001; GH3: 23.54 ± 1.25%, *p* = 0.002) (Figure 6A,B). In the TNF-α intervention assays, anti-TNF-α neutralization reduced shNC-CM-induced death in AtT-20 cells (32.55 ± 1.15% to 26.57 ± 1.19%, *p* = 0.003) and GH3 cells (30.92 ± 1.11% to 27.91 ± 1.29%, *p* = 0.038), while sh*Piezo1*-CM exposure showed a similar reduction relative to shNC-CM (AtT-20: 22.67 ± 1.21%, *p* < 0.001; GH3: 25.33 ± 1.20%, *p* = 0.004) (Figure 6C,D). Together with RIPK1/RIPK3/MLKL readouts, these findings support a TNF-α-associated necroptotic death program. TEM further showed nuclear morphological changes, cytoplasmic vacuolization, organelle injury, and plasma membrane rupture in shNC-CM-treated tumor cells, which were less evident after anti-TNF-α treatment or sh*Piezo1*-CM exposure (Figure 6E). Immunofluorescence staining and quantification showed increased p-RIPK1, p-RIPK3, and p-MLKL after TNF-α or shNC-CM treatment, whereas sh*Piezo1*-CM or GsMTx4 reduced these necroptosis-associated readouts (Figure 6F,G and Figure S6A,B). Western blotting further confirmed that shNC-CM increased p-RIPK1, p-RIPK3, and p-MLKL levels in both GH3 and AtT-20 cells, whereas sh*Piezo1*-CM or GsMTx4 reduced most of these necroptosis-associated readouts (Figure 6H,I and Figure S6C). In vivo, tumors from the sh*Piezo1*-RAW264.7 group contained lower p-RIPK1, p-RIPK3, and p-MLKL levels than those from the shNC-RAW264.7 group (Figure 6J,K and Figure S6D). Clinical IF and IHC analyses further detected increased p-MLKL, Caspase-1, and IL-1β signals in apoplectic tissues (Figure 6L–N and Figure S6E).

### 2.7. Pathological Necrosis Marks a Clinically Severe Apoplexy Phenotype

**Figure 7:**
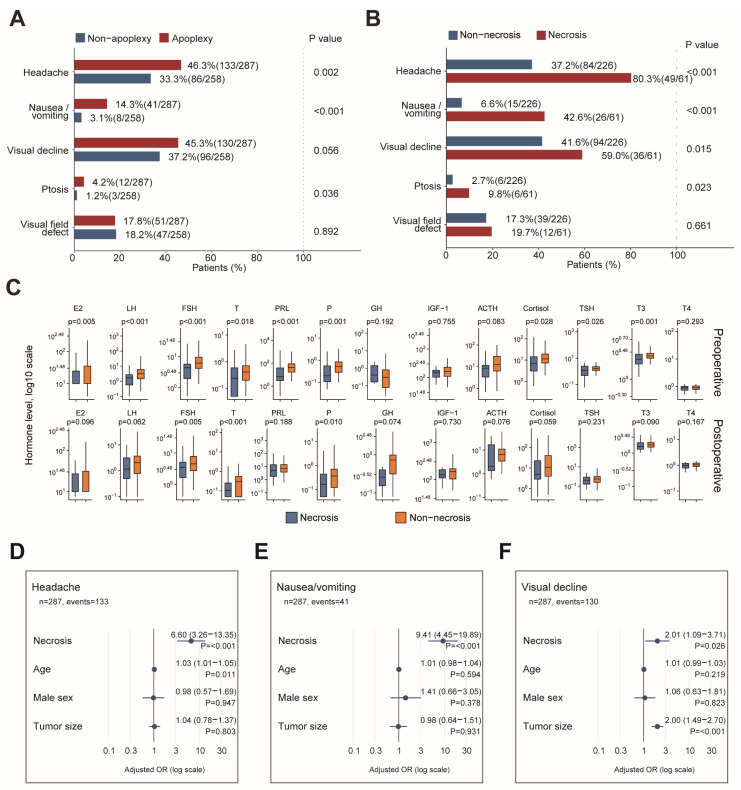
Pathological necrosis marks a clinically severe apoplexy phenotype. (**A**) Symptom-frequency comparison between non-apoplectic and apoplectic patients. (**B**) Symp-tom-frequency comparison between non-necrosis and necrosis subgroups within the apoplexy cohort. (**C**) Preoperative and postoperative pituitary hormone levels stratified by necrosis status. (**D**) Multivariable logistic regression forest plot for headache, adjusted for age, sex, and tumor size. (**E**) Multivariable logistic regression forest plot for nausea/vomiting, adjusted for age, sex, and tumor size. (**F**) Multivariable logistic regression forest plot for visual decline, adjusted for age, sex, and tumor size.

We next examined whether pathological necrosis was associated with clinical severity in pituitary apoplexy. Compared with non-apoplectic patients, patients with apoplexy more frequently presented with headache, nausea/vomiting, and ptosis, whereas visual decline and visual field defect did not differ significantly (Figure 7A). Within the apoplexy cohort, H&E-defined necrosis was associated with higher frequencies of headache (80.3% vs. 37.2%, *p* < 0.001), nausea/vomiting (42.6% vs. 6.6%, *p* < 0.001), visual decline (59.0% vs. 41.6%, *p* = 0.015), and ptosis (9.8% vs. 2.7%, *p* = 0.023) but not visual field defect (Figure 7B). Hormone profiling revealed necrosis-associated differences mainly involving the gonadal, thyroid, and ACTH–adrenal axes (Figure 7C and Figure S7A). After adjustment for age, sex, and tumor size, necrosis remained associated with headache (adjusted OR = 6.60, 95% CI: 3.26–13.35, *p* < 0.001), nausea/vomiting (adjusted OR = 9.41, 95% CI: 4.45–19.89, *p* < 0.001), and visual decline (adjusted OR = 2.01, 95% CI: 1.09–3.71, *p* = 0.026), while two or more clinical symptoms also remained strongly associated with necrosis (Figure 7D–F and Figure S7B–D).

## 3. Discussion

This study suggests that activated macrophages in apoplectic PitNETs may contribute to TNF-α-associated tumor cell necroptosis through PIEZO1-related signaling. PitNETs are not immunologically inert; macrophages are among the most consistently detected infiltrating immune cells and may shape tumor behavior [31,32]. Recent single-cell studies have highlighted macrophage–tumor interaction axes across several PitNET contexts [13,14,15], and spatial transcriptomic analyses have linked PitNET progression and invasion to immune remodeling [33,34]. In our dataset, the inferred communication network between tumor cells and macrophages was markedly strengthened in the apoplectic state. This pattern argues against a purely passive immune response and suggests that local inflammatory amplification may influence the extent and persistence of tissue injury after the initiating vascular event.

Macrophage analysis further indicated that apoplexy-associated macrophages were not simply an expanded uniform population. Instead, pseudotime, regulon, and histological evidence pointed to a continuum of inflammatory states shaped by activation, stress responses, and metabolic remodeling. Such plasticity is consistent with recent PitNET multi-omic studies and methodological reviews emphasizing that immune cells within PitNETs are dynamically conditioned by their local microenvironment rather than fixed in binary phenotypes [22,35,36].

The sellar compartment is anatomically constrained, and apoplexy may therefore develop in a setting of abnormal pressure and tissue tension. Within this context, PIEZO1 may provide a plausible mechanosensitive link to macrophage reprogramming. However, direct evidence that increased intrasellar pressure activates this pathway in situ is not yet available. PIEZO1 participates in the macrophage sensing of matrix stiffness and mechanical stimulation and can regulate macrophage functional output [18,37,38]. More broadly, PIEZO channels convert mechanical cues into Ca^2+^-dependent intracellular responses, placing them at the interface between tissue mechanics and inflammation [38,39,40]. This relationship is not universal or linear: PIEZO1 signaling varies by cell type, tissue context, and immune state [41,42,43]. Accordingly, PIEZO1 should be viewed as a context-dependent transducer of local mechanical perturbation, rather than as a simple pro-inflammatory switch. In our in vitro experiments, Yoda1-mediated PIEZO1 activation increased calcium-associated fluorescence, promoted NFATC2/REL nuclear enrichment, and induced *Tnf*, *Il1b*, and *Il6* mRNA expression in macrophages; *Piezo1* silencing or Ca^2+^ chelation weakened these responses. The findings align with prior work linking PIEZO1 to macrophage mechanosensing and Ca^2+^-dependent inflammatory regulation [18,22,40], while the known roles of NFATC2 and NF-κB/REL in macrophage inflammation further support this interpretation [43,44]. Models that preserve the in situ mechanical and cellular architecture will be important for testing this mechanism more directly.

Inflammatory mediators such as TNF-α and IFN-γ can activate RIPK1/RIPK3/MLKL-dependent necroptosis and may intersect with other regulated cell death programs [45,46]. In the present study, the tumor cell death phenotype was interpreted as necroptosis because it was accompanied by increased p-RIPK1, p-RIPK3, and p-MLKL and was reduced when macrophage Piezo1-related signaling was inhibited. Consistently, the suppression of the macrophage PIEZO1/Ca^2+^/NFATC2/REL axis lowered TNF-α levels in conditioned medium and attenuated tumor cell death. Clinical stratification added a translational layer: pathological necrosis in patients with pituitary apoplexy was associated with more frequent acute symptoms and selected perioperative hormonal abnormalities. These observations do not prove that necrosis alone drives the clinical syndrome, but they suggest that H&E-defined necrosis marks a biologically and clinically more severe apoplexy state.

Several limitations should be noted. First, single-cell sequencing and clinicopathological analyses provide cross-sectional evidence and cannot reconstruct the temporal sequence before and after apoplexy onset. Second, the in vitro experiments relied on cell lines and conditioned medium systems, which cannot fully reproduce the native human PitNET microenvironment. Third, although the data support a close relationship between the PIEZO1–Ca^2+^–NFATC2/REL–TNF axis and apoplexy-associated inflammatory responses, they do not directly establish that increased intrasellar pressure activates this axis in situ. Finally, macrophage-derived mediators other than TNF may also contribute to tumor cell death. Future studies using additional macrophage models (e.g., human THP-1-derived or bone marrow-derived macrophages), primary cells, organoid or explant systems, controlled mechanical stimulation, and direct assays of necrosome formation will help refine this model.

## 4. Materials and Methods

### 4.1. Clinical Specimens and Clinical Data

Human PitNET specimens and matched clinical data were obtained from patients who underwent transsphenoidal surgery at the Department of Neurosurgery, Tongji Hospital, Tongji Medical College, Huazhong University of Science and Technology. This single-center study integrated clinical specimens with mechanistic experiments. The clinical apoplexy cohort initially included 292 surgically treated patients diagnosed with pituitary apoplexy between January 2012 and January 2022. Referring to Figure 7, 287 patients with complete symptom records, necrosis status, age, sex, and tumor size data were included in the apoplexy symptom frequency analysis, necrosis-stratified analyses, and multivariable logistic regression. The non-apoplectic control cohort included 258 surgically treated non-apoplectic PitNET patients between January 2022 and January 2025 who served as controls for the symptom frequency analysis in Figure 7A. Fresh specimens for scRNA-seq, primary cell preparation, or histological validation were transferred to the laboratory immediately after resection.

This study was conducted in accordance with the Declaration of Helsinki and approved by the Ethics Committee of Tongji Hospital, Tongji Medical College, Huazhong University of Science and Technology (TJ-IRB20220325). Written informed consent was obtained from all participants or their legal representatives. A retrospective analysis of anonymized clinical data was performed under the approved protocol.

Eligible patients had a postoperative pathological diagnosis of PitNET; available tumor tissue for histological, molecular, or cellular analyses; and basic clinical information, including symptoms, imaging findings, operative records, and pathological diagnosis. Patients were excluded if they had prior pituitary surgery or radiotherapy, uncertain pathological diagnosis, severely incomplete clinical data, tissue of insufficient quality for the planned analysis, or concomitant disease that could confound the pathological assessment of the pituitary region. Fresh tissues for scRNA-seq were placed in ice-cold sterile preservation solution immediately after resection and processed for mechanical dissociation, enzymatic digestion, and single-cell suspension preparation.

Pituitary apoplexy was diagnosed using acute or subacute symptoms, including headache, nausea/vomiting, visual decline, visual field defect, and ptosis, together with imaging evidence of intratumoral hemorrhage and/or infarction and postoperative H&E-based pathological assessment. Non-apoplectic PitNETs had no clinical, radiological, or pathological evidence of apoplexy. Pathological necrosis was defined as identifiable coagulative necrosis, infarct-like change, or extensive disruption of cellular architecture on H&E-stained sections; no fixed necrotic area threshold was applied. Patients with pituitary apoplexy were stratified into Necrosis and Non-necrosis groups according to the presence or absence of definite H&E-defined necrosis. Necrotic regions were used for histological localization and image analysis.

Demographic information, tumor size, Ki-67 index, clinical symptoms, operative records, imaging reports, pathological diagnoses, and perioperative hormone data were retrieved from hospital medical record and pathology systems. The analyzed symptoms were headache, nausea/vomiting, visual decline, visual field defect, and ptosis. Each symptom was encoded as a binary variable based on the medical record. The variable for two or more clinical symptoms was defined as the presence of any two or more of these five symptoms. Multivariable logistic regression in Figure 7D–F and Supplementary Figure S7B–D was restricted to the apoplexy cohort and used pathological necrosis as the main explanatory variable. Hormone data were grouped by pituitary axes, including the gonadal, thyroid, and ACTH–adrenal axes. Preoperative hormone levels were the most recent morning fasting measurements obtained after admission and before surgery; postoperative hormone levels were obtained at the corresponding postoperative follow-up time points. The results were interpreted according to contemporaneous reference ranges from the Clinical Laboratory Center of Tongji Hospital.

### 4.2. Histopathological Assessment

Tumor tissues were fixed in 4% paraformaldehyde (Servicebio, Wuhan, China) for 24–48 h, dehydrated, embedded in paraffin, and serially sectioned at 5 µm. H&E staining was used to assess hemorrhage, necrosis, inflammatory cell infiltration, and tumor morphology. The Ki-67 index and pathological subtype were obtained from clinical pathology reports. When required, two pathologists with neuropathology experience re-evaluated hotspot regions. The Ki-67 index was reported as the percentage of immunopositive tumor cells, preferentially counted in regions with the highest proliferative activity.

Necrotic regions were identified on H&E-stained sections and spatially matched with adjacent serial sections or immunofluorescence images from the same region. Serial sections were aligned according to tissue architecture, vascular orientation, hemorrhagic borders, and necrotic margins. Two observers blinded to experimental grouping independently performed pathological evaluation, and discrepant findings were resolved by joint review.

### 4.3. Immunohistochemistry and Immunofluorescence

Paraffin sections were deparaffinized in xylene, rehydrated through graded ethanol, and subjected to antigen retrieval. EDTA antigen retrieval buffer (Servicebio, Wuhan, China; G1203, pH 8.0–9.0) or citrate antigen retrieval buffer (Servicebio, Wuhan, China; pH 6.0) was applied at 95–100 °C for 15–20 min according to antibody requirements and preliminary optimization. For immunohistochemistry (IHC), endogenous peroxidase activity was blocked with 3% H_2_O_2_ for 10–15 min at room temperature. Sections were blocked with 5% bovine serum albumin (BSA; Servicebio, Wuhan, China; GC305006-100 g) or normal goat serum (Servicebio, Wuhan, China) for 1 h and incubated with primary antibodies overnight at 4 °C. After PBS/PBST washing, corresponding HRP-conjugated or biotinylated secondary antibodies were applied at room temperature, followed by SABC/DAB development (Gene Technology [Shanghai] Co., Ltd., Shanghai, China; GK600510), hematoxylin counterstaining, dehydration, clearing, and mounting.

For immunofluorescence (IF), sections were incubated with primary antibodies against IBA-1/Iba1, HLA-DR, CD206, PIEZO1/Piezo1, TNF-α, p-MLKL, Caspase-1, and IL-1β after antigen retrieval and blocking. Sections were then incubated with Alexa Fluor 488-conjugated IgG (Abcam, Cambridge, UK; ab150077/ab150113), Cy3-conjugated IgG (Proteintech Group, Rosemont, IL, USA; SA00009-2/SA00009-1), or fluorescent secondary antibodies from the TSA Plus kit (Servicebio, Wuhan, China) for 1–2 h at room temperature in the dark, followed by DAPI nuclear counterstaining (Servicebio, Wuhan, China) for 10–15 min. Multiplex IF was preferentially performed by sequential staining with primary antibodies from different host species. If host species overlapped or signals were weak, TSA amplification was used. Staining, washing, and image acquisition parameters were kept consistent within each experimental batch. Antibody details are provided in Supplementary Table S1.

### 4.4. Image Acquisition and Quantitative Analysis

Bright-field and fluorescence images were acquired using an Olympus bright-field microscope (Olympus Corporation, Tokyo, Japan) and a CKX53 fluorescence microscope (Olympus Corporation, Tokyo, Japan). Whole-slide scanned images were acquired using a PANNORAMIC series digital slide scanner (3DHISTECH Ltd., Budapest, Hungary) and viewed using CaseViewer software (version 2.4; 3DHISTECH Ltd., Budapest, Hungary). For group comparisons, exposure time, laser intensity, gain, white balance, and threshold settings were held constant within the same staining batch. Five non-overlapping high-power fields or five matched regions of interest were selected for each sample. Necrotic borders and adjacent regions were compared using regions of interest with identical areas. Observers blinded to group allocation performed image analysis.

Image quantification was performed using ImageJ/Fiji software (version 1.53 or bundled 64-bit Java 8 version). IBA-1-positive cells were quantified as positive cells per high-power field, relative positive area, or immunoreactive area. PIEZO1 and p-MLKL signals were quantified as mean fluorescence intensity. Caspase-1 and IL-1β IHC signals were quantified as integrated optical density or the mean gray value. TUNEL positivity was calculated as TUNEL-positive nuclei divided by DAPI-positive nuclei multiplied by 100%. For gray value line profiles, identical paths were drawn across IBA-1-positive and TNF-α-positive regions, and fluorescence intensity was extracted from the corresponding channels. Pearson correlation was used to assess the association between IBA-1 and TNF-α signals; Spearman correlation was used when data were not normally distributed.

### 4.5. Single-Cell RNA Sequencing and Data Processing

Fresh PitNET tissues were minced under sterile conditions into approximately 1 mm^3^ fragments and digested in collagenase IV, dispase II, and DNase I at 37 °C with gentle agitation for 30–45 min. During digestion, suspensions were gently pipetted every 5–10 min to improve cell release and reduce mechanical damage. Digestion was stopped, and the suspension was sequentially filtered through 70 µm and 40 µm cell strainers (Servicebio, Wuhan, China). After PBS washing (Servicebio, Wuhan, China), red blood cells were lysed when necessary using red blood cell lysis buffer (Servicebio, Wuhan, China). Cell viability was assessed by trypan blue exclusion, and samples with viability > 80% were used for library construction.

Single-cell libraries were prepared using the 10× Genomics Chromium Single Cell 3′ platform (10× Genomics, Pleasanton, CA, USA) and Chromium Next GEM Single Cell 3′ Reagent Kit v3.1 (10× Genomics, Pleasanton, CA, USA). After quality control, libraries underwent paired-end sequencing on an Illumina NovaSeq platform (Illumina, San Diego, CA, USA). Raw data were processed with Cell Ranger (version 10.0.0; 10× Genomics, Pleasanton, CA, USA) and aligned to the GRCh38 human reference genome. Downstream analyses were performed in R (version v4.2.0) and Python (version 3.8.13).

Quality control, normalization, dimensionality reduction, and clustering were performed mainly with Seurat (version 5.4.0). Cells were retained when nFeature_RNA was 200–6000, percent.mt was <20%, nCount_RNA was within an appropriate range, and doublet risk was low. Obvious doublets and low-quality cells were removed using DoubletFinder (version 2.0.6) or an equivalent method. The standard workflow included NormalizeData, FindVariableFeatures, ScaleData, RunPCA, RunUMAP, and FindClusters. Harmony (version 1.2.4) was used for batch correction when required. Cell types were annotated using canonical markers, reference annotations, and published PitNET single-cell features, including PitNET tumor cells, macrophages, T and natural killer (NK) cells, endothelial cells, pericytes/vascular smooth muscle cells (vSMCs), and proliferating/cycling cells.

Differential gene expression was assessed using the Wilcoxon rank-sum test or an equivalent Seurat-supported method, with Benjamini–Hochberg correction for multiple testing. Unless stated otherwise, adjusted *p* < 0.05 and |log2 fold change| > 0.25 were used as screening thresholds. Pathway analyses included Hallmark gene sets, Gene Ontology, KEGG, and GSEA, implemented mainly using clusterProfiler (version 4.18.4). Macrophages were reclustered into M0–M7 subclusters. Pseudotime analysis was performed with Monocle3 (version 1.4.26), with the root state defined by a low-inflammatory, low-activation macrophage state and trajectory structure. Cell–cell communication was analyzed using CellChat (R package, version 1.6.1) to compare interaction number, interaction strength, incoming/outgoing signaling patterns, and relative information flow between groups.

### 4.6. Regulon, Motif, and Promoter Analyses

Macrophage regulon activity was inferred using the pySCENIC workflow (version 0.12.1). Gene regulatory networks were constructed with GRNBoost2, followed by motif enrichment in candidate regulons using the cisTarget motif database. Single-cell regulon activity was calculated with AUCell. Macrophage state-associated transcriptional regulators were prioritized according to regulon specificity scores and a binarized activity matrix. NFATC2 and REL/c-Rel were selected for mechanistic analysis because of their activity in inflammation-associated macrophage states.

Correlations of *PIEZO1*/*PIEZO2* with *NFATC2*, *REL*, *TNF*, *NFKB1*, *IL1B*, and *RELA* were calculated at the single-cell level within macrophages and at the pseudobulk level after aggregation by sample or subcluster. Spearman correlation was used, followed by Benjamini–Hochberg correction for multiple testing. REL/c-Rel and RELA/p65 are treated as distinct NF-κB family members throughout this manuscript.

Motif analysis was performed using the JASPAR 2024 CORE vertebrate database, with NFATC2 represented by matrix MA0152.1 (TTTTCCA, 7 bp) and REL/c-Rel by matrix MA0101.1 (GGGGATTTCC, 10 bp). Promoters were defined as 2 kb upstream to 500 bp downstream of the transcription start site. Human, mouse, and rat sequences were extracted from the GRCh38, GRCm39/mm39, and mRatBN7.2/rn7 reference genomes, respectively. Candidate NFATC2 and REL/c-Rel motifs in the *TNF*/*Tnf* and *NFKBIA*/*Nfkbia* promoters guided ChIP-qPCR site design.

### 4.7. Cell Culture

RAW264.7 macrophages and GH3, AtT-20, and HEK293T cells were used for in vitro experiments. RAW264.7, GH3, AtT-20, and 293T cells were obtained from ATCC (Manassas, VA, USA) or ATCC-authenticated repositories; commonly used catalog numbers are RAW264.7 (TIB-71), GH3 (CCL-82.1), AtT-20 (CCL-89), and 293T (CRL-3216). Cells were maintained in DMEM/High-Glucose medium (Servicebio, Wuhan, China) containing 10% fetal bovine serum (Abbkine Scientific Co., Ltd., Wuhan, China) and 1% penicillin–streptomycin (Servicebio, Wuhan, China) at 37 °C in 5% CO_2_. Cells were passaged at 70–80% confluence, and passages 3–20 after thawing were used for functional experiments.

*Mycoplasma* contamination was tested monthly or after thawing or viral infection and before key functional experiments using a Mycoplasma Stain Assay Kit (Beyotime Biotechnology, Shanghai, China; C0296). HEK293T cells for lentiviral packaging were maintained in complete DMEM/High-Glucose medium and seeded one day before transfection to reach 60–80% confluence.

### 4.8. Macrophage Activation and Piezo1 Manipulation

PIEZO1 activity in RAW264.7 macrophages was pharmacologically activated with Yoda1 and inhibited with GsMTx4 when required. Yoda1, GsMTx4, VIVIT, and IT-901 were purchased from MedChemExpress (Monmouth Junction, NJ, USA). Yoda1 was dissolved in DMSO (Servicebio, Wuhan, China) and used at 10 µM for routine 24 h stimulation. For Ca^2+^ fluorescence assays, images were acquired 120 s after Yoda1 addition. GsMTx4 was dissolved in sterile water or PBS and applied at 5 µM for 30–60 min before Yoda1 stimulation. VIVIT was used at 10 µM and IT-901 at 3 µM; both were added 1 h before Yoda1 stimulation. Vehicle-treated cells served as controls, and the final DMSO concentration was kept below 0.1% in all groups.

*Piezo1* knockdown was performed by shRNA-mediated lentiviral silencing. shRNA sequences (Supplementary Table S2) were designed and synthesized by Tsingke Biotechnology Co., Ltd. (Beijing, China) and cloned into the pLKO.1 lentiviral vector; a non-targeting shRNA was used as the negative control. Target plasmids were co-transfected with psPAX2 and pMD2.G packaging plasmids (Tsingke Biotechnology Co., Ltd., Beijing, China) into HEK293T cells using Lipofectamine 3000 (Invitrogen/Thermo Fisher Scientific, Waltham, MA, USA; L3000015). Viral supernatants were collected at 48 and 72 h, filtered through 0.45 µm or 0.22 µm filters (MilliporeSigma, Burlington, MA, USA), and used to infect RAW264.7 cells in the presence of polybrene (8 µg/mL). Stable knockdown cells were selected with puromycin (MedChemExpress, Monmouth Junction, NJ, USA; 2–5 µg/mL) for 5–7 days. *Piezo1* knockdown efficiency was verified by RT-qPCR and/or Western blot before functional experiments.

For inflammatory activation experiments, RAW264.7 cells were treated with Yoda1 (MedChemExpress [MCE]; HY-18723; 10 µM) in the presence or absence of *Piezo1* knockdown, GsMTx4 (MCE; HY-P1410; 5 µM), VIVIT (MCE; HY-P1430A; 10 µM), or IT-901 (MCE; HY-124179; 3 µM). Cells were collected for mRNA or protein analysis of *Nos2*/iNOS, *Tnf*/TNF-α, *Il1b*/IL-1β, and *Il6*/IL-6, and culture supernatants were collected for cytokine measurements. Group names followed the corresponding figures, including shNC_vehicle, shNC_Yoda1, sh*Piezo1*_Yoda1, shNC_Yoda1_GsMTx4, Ctrl_NC, Ctrl_sh, Yoda1_NC, and Yoda1_sh.

### 4.9. Preparation of Macrophage-Conditioned Medium and Tumor Cell Treatment

Conditioned medium (CM) was prepared from RAW264.7 macrophages seeded at 2 × 10^5^ cells/well in 6-well plates (Corning Inc., Corning, NY, USA). After attachment, cells received shNC, sh*Piezo1*, Yoda1 stimulation, and TNF-α-related interventions according to the experimental design. To reduce serum background, medium was replaced with DMEM/High-Glucose medium containing 2% FBS before CM collection, and cells were treated for 24 h. Supernatants were collected, centrifuged at 1000× *g* for 5 min at 4 °C to remove suspended cells and again at 3000× *g* for 10 min to remove debris, then filtered through a 0.22 µm sterile filter (MilliporeSigma, Burlington, MA, USA). CM was used fresh whenever possible; aliquots were stored at −80 °C when necessary, avoiding repeated freeze–thaw cycles.

GH3 and AtT-20 cells were seeded at 1 × 10^5^ cells/well in 6-well plates or at equivalent proportions in other culture formats. Macrophage-derived CM was mixed with fresh complete medium at a 1:1 ratio and applied to GH3 or AtT-20 cells for 24–48 h. Direct Yoda1 treatment and CM treatment were analyzed separately to distinguish direct drug effects from macrophage-mediated effects.

To assess TNF-α contribution, mouse recombinant TNF-α (ABclonal Technology, Wuhan, China; 20 ng/mL) was added to sh*Piezo1*-CM, whereas a TNF-α neutralizing antibody (ABclonal Technology, Wuhan, China; 1 µg/mL) was added to shNC-CM and preincubated at 37 °C for 1 h before tumor cell treatment. Tumor cell death or necroptosis-associated injury was then assessed by Annexin V/PI flow cytometry and transmission electron microscopy.

### 4.10. Flow Cytometric Analysis of Cell Death

GH3 and AtT-20 cells were collected after treatment, washed twice with cold PBS, and stained using an Annexin V-FITC/PI apoptosis assay kit (Yeasen Biotechnology, Shanghai, China; 40302ES) according to the manufacturer’s protocol. Briefly, 1 × 10^5^ to 1 × 10^6^ cells per sample were resuspended in binding buffer, incubated with Annexin V-FITC and PI for 15 min at room temperature in the dark, diluted with binding buffer, and analyzed within 1 h. At least 10,000 valid events were collected per sample using a CytoFLEX flow cytometer (Beckman Coulter, Brea, CA, USA) or equivalent instrument. Data were analyzed in FlowJo v10.8 software (BD Biosciences, Ashland, OR, USA) or equivalent software. Viable, early apoptotic, late apoptotic, and PI-positive dead/necrotic populations were defined by Annexin V and PI signals. Final quantification included Annexin V-positive, PI-positive, or Annexin V/PI double-positive cells according to the definitions used in each figure.

### 4.11. TUNEL Assay and Transmission Electron Microscopy

TUNEL staining assessed DNA fragmentation in clinical tissue sections. After deparaffinization and rehydration, sections were permeabilized with Proteinase K (Servicebio, Wuhan, China; G1234) for 10–20 min, washed with PBS, and incubated with a TUNEL apoptosis detection kit (Servicebio, Wuhan, China; G1501) at 37 °C for 1 h in the dark. Nuclei were counterstained with DAPI (Servicebio, Wuhan, China), and images were acquired using identical settings within each batch. TUNEL positivity was calculated as TUNEL-positive nuclei divided by DAPI-positive nuclei multiplied by 100%.

Transmission electron microscopy (TEM) was used to examine ultrastructural changes in treated tumor cells. Cells were collected, fixed with electron microscopy fixative (Servicebio, Wuhan, China) at 4 °C, postfixed with osmium tetroxide, dehydrated through graded ethanol or acetone, embedded in epoxy resin, sectioned into 60–80 nm ultrathin slices, and stained with uranyl acetate and lead citrate. Samples were observed using an HT7800 transmission electron microscope (Hitachi High-Tech Corporation, Tokyo, Japan). The ultrastructural features recorded included plasma membrane integrity, organelle swelling, mitochondrial crista changes, chromatin alterations, and cell rupture.

### 4.12. RT-qPCR

Total RNA was extracted from cultured macrophages or tumor cells using TRIzol reagent (Servicebio, Wuhan, China; G3013) or an equivalent reagent. RNA concentration and purity were determined by spectrophotometry. For each sample, 1 µg total RNA was reverse-transcribed into cDNA using a reverse transcription reagent kit (Yeasen Biotechnology, Shanghai, China; 11141ES).

qPCR was performed using Hieff qPCR SYBR Green Master Mix (Yeasen Biotechnology, Shanghai, China; 11202ES) on a QuantStudio 1 Real-Time PCR System (Thermo Fisher Scientific, Waltham, MA, USA) or an equivalent instrument. Cycling conditions were 95 °C for 5 min, followed by 40 cycles of 95 °C for 10 s and 60 °C for 30 s. Relative mRNA expression was calculated using the 2^-ΔΔCt method, with *Gapdh*/GAPDH as the internal control. Primer sequences for *Piezo1*, *Nos2*/iNOS, *Tnf*/TNF-α, *Il1b*/IL-1β, *Il6*/IL-6, and other target genes are listed in Supplementary Table S3.

### 4.13. Cytokine Measurements

Cytokines in macrophage culture supernatants were measured using ELISA kits (Elabscience Biotechnology Co., Ltd., Wuhan, China). The analyzed cytokines included IL-1β, TNF-α, IL-6, IFN-γ, IL-12P40, IL-12P70, CCL2, CCL5, CXCL1, GM-CSF, IL-4, and IL-10, according to the markers shown in the corresponding figures.

After treatment, supernatants were collected and centrifuged at 12,000× *g* for 10 min at 4 °C to remove debris. Samples were analyzed immediately or aliquoted and stored at −80 °C, avoiding repeated freeze–thaw cycles. ELISAs followed the manufacturer’s instructions and used standard curves. Samples outside the detection range were diluted and reanalyzed. At least three biological replicates were included in each group, with technical duplicates or triplicates for each sample.

### 4.14. Western Blotting and Nuclear–Cytoplasmic Fractionation

Cells or tissue samples were lysed in RIPA lysis buffer (Servicebio, Wuhan, China; G2002) supplemented with protease inhibitor cocktail (Servicebio, Wuhan, China; G2008) and phosphatase inhibitor cocktail (Servicebio, Wuhan, China; G2007). Lysis was performed on ice for 30 min, and supernatants were collected by centrifugation. Protein concentration was measured using a BCA protein assay kit (Servicebio, Wuhan, China). For each sample, 20–30 µg protein was separated on SDS-PAGE gels (Servicebio, Wuhan, China; G2037) and transferred to PVDF membranes (MilliporeSigma, Burlington, MA, USA; IPVH00010/IPFL00005).

Membranes were blocked with 5% non-fat milk, 5% BSA, or NcmBlot blocking buffer (NCM Biotech, Suzhou, China; P30500) for 1 h at room temperature and incubated overnight at 4 °C with primary antibodies against NFATC2, REL/c-Rel, p-RIPK1, RIPK1, p-RIPK3, RIPK3, p-MLKL, MLKL, and loading controls including β-tubulin and Lamin B1. After TBST washing, HRP-conjugated secondary antibodies (Proteintech Group, Rosemont, IL, USA; SA00001-1/SA00001-2) were applied for 1–2 h at room temperature. Bands were visualized using NcmECL Ultra (NCM Biotech, Suzhou, China; P10300) and captured with a ChemiDoc XRS+ System (Bio-Rad Laboratories, Hercules, CA, USA). Band intensity was quantified in ImageJ/Fiji and normalized to the appropriate loading controls.

For nuclear–cytoplasmic fractionation, RAW264.7 macrophages were treated as indicated, and nuclear and cytoplasmic protein fractions were obtained using a Nuclear and Cytoplasmic Protein Extraction Kit (Beyotime Biotechnology, Shanghai, China; P0027) or a validated fractionation protocol. NFATC2 and REL/c-Rel nuclear translocation was assessed using Lamin B1 as the nuclear loading control and β-tubulin or GAPDH as the cytoplasmic loading control. When cropped Western blots are shown in the main figures, complete membrane images, molecular weight markers, and lane/loading order are provided in the Supplementary Materials.

### 4.15. Calcium Fluorescence Assay

Intracellular Ca^2+^ levels in RAW264.7 macrophages were assessed using Calcium 520 AM (KKL Med Inc., Ashland, VA, USA; KM31725). Cells were seeded on glass-bottom dishes or coverslips and loaded with 5 µM Calcium 520 AM in Ca^2+^/Mg^2+^-containing HBSS (Gibco/Thermo Fisher Scientific, Waltham, MA, USA) supplemented with 0.02% Pluronic F-127 (Thermo Fisher Scientific, Waltham, MA, USA). Cells were loaded at 37 °C for 30 min in the dark, washed three times with HBSS, and de-esterified for 20–30 min at 37 °C or room temperature.

Yoda1 was added to a final concentration of 10 µM according to the experimental grouping. Calcium 520 fluorescence images were acquired 120 s after Yoda1 treatment using identical microscope settings. Exposure time, gain, and acquisition conditions were kept constant across groups. Fluorescence intensity was quantified in ImageJ/Fiji and expressed as mean or relative fluorescence intensity. At least three independent experiments were performed for each group, with effective cells analyzed from multiple fields in each experiment.

### 4.16. ChIP-qPCR

ChIP-qPCR was used to assess NFATC2 and REL/c-Rel enrichment at candidate promoter regions. RAW264.7 macrophages were treated as indicated and cross-linked with 1% formaldehyde for 10 min at room temperature. Cross-linking was quenched with glycine at a final concentration of 125 mM for 5 min. Cells were then washed, lysed, and sonicated to shear chromatin into 200–500 bp fragments.

Sheared chromatin was incubated overnight with anti-NFATC2 (Santa Cruz Biotechnology, Dallas, TX, USA; sc-7296 X), anti-REL/c-Rel (Santa Cruz Biotechnology, Dallas, TX, USA; sc-6955 X), or normal IgG (Santa Cruz Biotechnology, Dallas, TX, USA; sc-2025). Immunocomplexes were captured with Protein A/G Magnetic Beads (MedChemExpress, Monmouth Junction, NJ, USA; HY-K0202). After sequential washing with low-salt, high-salt, LiCl, and TE buffers, complexes were eluted and reverse-cross-linked at 65 °C. DNA was purified and analyzed by qPCR.

qPCR primers covered predicted NFATC2/REL binding sites and a negative control region in the *Tnf* promoter. The experimentally used *Tnf* promoter amplicon included the mm39 chr17:35421683-35421763 region. Other candidate sites, negative regions, and *Il6* and *Il1b* promoter-associated sites are listed in Supplementary Table S3. Enrichment was expressed as the percentage of input and fold enrichment over IgG.

### 4.17. Animal Experiments

Animal experiments were performed to determine whether *Piezo1* knockdown in macrophages modulates necroptosis-associated signaling in pituitary tumor tissues. Six-week-old male BALB/c nude mice were purchased from GemPharmatech Co., Ltd. (Nanjing, China) and housed under specific pathogen-free conditions at the Experimental Animal Center of Tongji Medical College, Huazhong University of Science and Technology. All animal procedures were approved by the Animal Ethics Committee of Tongji Medical College, Huazhong University of Science and Technology (approval no. TJH-202206015) and were conducted in accordance with institutional animal care guidelines and the 3Rs principle. To establish the pituitary tumor xenograft model, GH3 cells were suspended in a 1:1 mixture of PBS (Servicebio, Wuhan, China) and Matrigel Matrix (Corning Inc., Corning, NY, USA; 356234) and injected subcutaneously into the axillary region of each mouse. Each mouse received 1 × 10^7^ GH3 cells. On day 21 after tumor cell implantation, mice bearing tumors of comparable size were randomly assigned to the shNC-RAW264.7 group or the sh*Piezo1*-RAW264.7 group. RAW264.7 macrophages transduced with shNC or sh*Piezo1* were then injected into the established tumors. Tumor tissues were collected on day 28 after tumor cell implantation. At the experimental endpoint, mice were euthanized, and tumor tissues were immediately harvested for a Western blot analysis of necroptosis-associated proteins, including p-RIPK1, p-RIPK3, and p-MLKL. Additional details regarding animal allocation, randomization, blinding, inclusion and exclusion criteria, animal monitoring, humane endpoints, experimental timeline, and endpoint assessment are provided in the Supplementary Animal Experiments Attachment.

### 4.18. Statistical Analysis

Statistical analyses were performed using GraphPad Prism 9.3 and R 4.2.0. Unless otherwise stated in the figure legends, data are presented as the mean ± SD. Data distribution was assessed with the Shapiro–Wilk test when sample size allowed. For two-group comparisons, normally distributed data were analyzed using unpaired or paired Student’s t-test, whereas non-normally distributed data were analyzed using the Mann–Whitney U test or Wilcoxon signed-rank test. For multiple-group comparisons, a one-way or two-way ANOVA followed by Tukey, Dunnett, or Sidak post hoc tests was used when parametric assumptions were met; otherwise, the Kruskal–Wallis test followed by Dunn’s multiple-comparisons test was applied.

Categorical variables were analyzed using the chi-square test or Fisher’s exact test. Correlations were assessed using Pearson or Spearman correlation according to data distribution. Multiple testing correction for single-cell differential analysis, pathway enrichment, and regulon/motif-related analyses used the Benjamini–Hochberg method. All statistical tests were two-sided, and *p* < 0.05 was considered statistically significant.

Multivariable logistic regression evaluated the association between pathological necrosis and clinical symptoms among patients with pituitary apoplexy. Each clinical symptom was modeled as a binary dependent variable, with necrosis as the main independent variable. Models were adjusted for age, sex, and tumor size. Sex was encoded as male sex, and tumor size was included as a continuous variable. The results are reported as adjusted odds ratios (adjusted ORs) with 95% confidence intervals (95% CIs). Figure 7D–F and Supplementary Figure S7B–D correspond to the logistic regression results for headache, nausea/vomiting, visual decline, visual field defect, ptosis, and two or more clinical symptoms, respectively.

## 5. Conclusions

In conclusion, compared with non-apoplectic tumors, apoplectic PitNETs exhibited a remodeled immune microenvironment characterized by macrophage accumulation, inflammatory activation, and enhanced cell death signaling. In vitro, PIEZO1 activation in macrophages was associated with Ca^2+^-dependent inflammatory signaling, and macrophage-conditioned medium promoted TNF-α-associated tumor cell necroptosis. Clinically, pathological necrosis was associated with a greater acute symptom burden and selected perioperative pituitary hormone abnormalities. Future studies are needed to obtain direct in situ evidence for this PIEZO1-related macrophage inflammatory axis and to determine whether it may serve as a potential target for intervention.

## Data Availability

The data generated in this study are available from the corresponding authors upon reasonable request.

## Supplementary Materials

The following supporting information can be downloaded at https://www.mdpi.com/article/10.3390/ijms27125635/s1.
