# Supplementary material for: Activated Macrophages Promote TNF-α-Associated Tumor Cell Necroptosis in Pituitary Apoplexy Through the PIEZO1–NFATC2/REL Axis
Source: Int J Mol Sci. 2026 Jun 22;27(12):5635. doi: 10.3390/ijms27125635 (PMC13299367; doi:10.3390/ijms27125635)
Supplement: Supplementary file 1 [file ijms-27-05635-s001.zip › ijms-4342468-supplementary.pdf]

Supplementary Figures

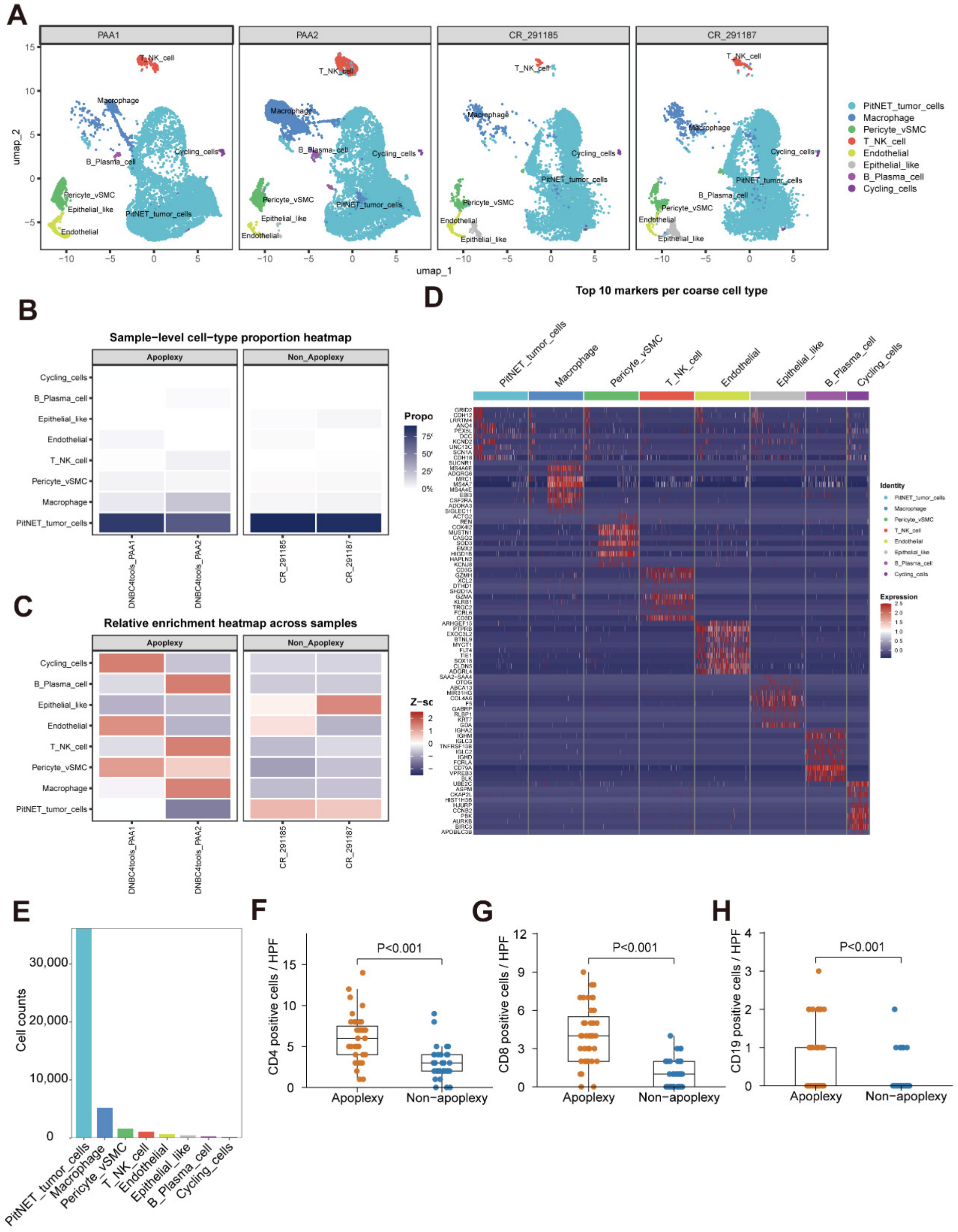

Supplementary Figure S1. Sample-level cell composition and histological validation of immune-cell infiltration in apoplectic and non-apoplectic PitNETs. (A) Sample-level UMAPs showing the distribution of cells from each PitNET specimen after integration. (B) Heatmap summarizing the relative proportions of annotated major cell populations across individual samples. (C) Cell-type enrichment heatmap showing the relative distribution of major cell populations between apoplectic and non-apoplectic samples. (D) Marker-gene heatmap supporting annotation of the major cell populations, including PitNET tumor cells,

macrophages, pericytes/vascular smooth muscle cells (vSMCs), T and natural killer (NK) cells, endothelial cells, epithelial-like cells, B and plasma cells, and proliferating/cycling cells. (E) Absolute cell counts of annotated cell populations in apoplectic and non-apoplectic samples. (F-H) Quantification of CD4-positive, CD8-positive, and CD19-positive cells in apoplectic and non-apoplectic PitNET tissues.

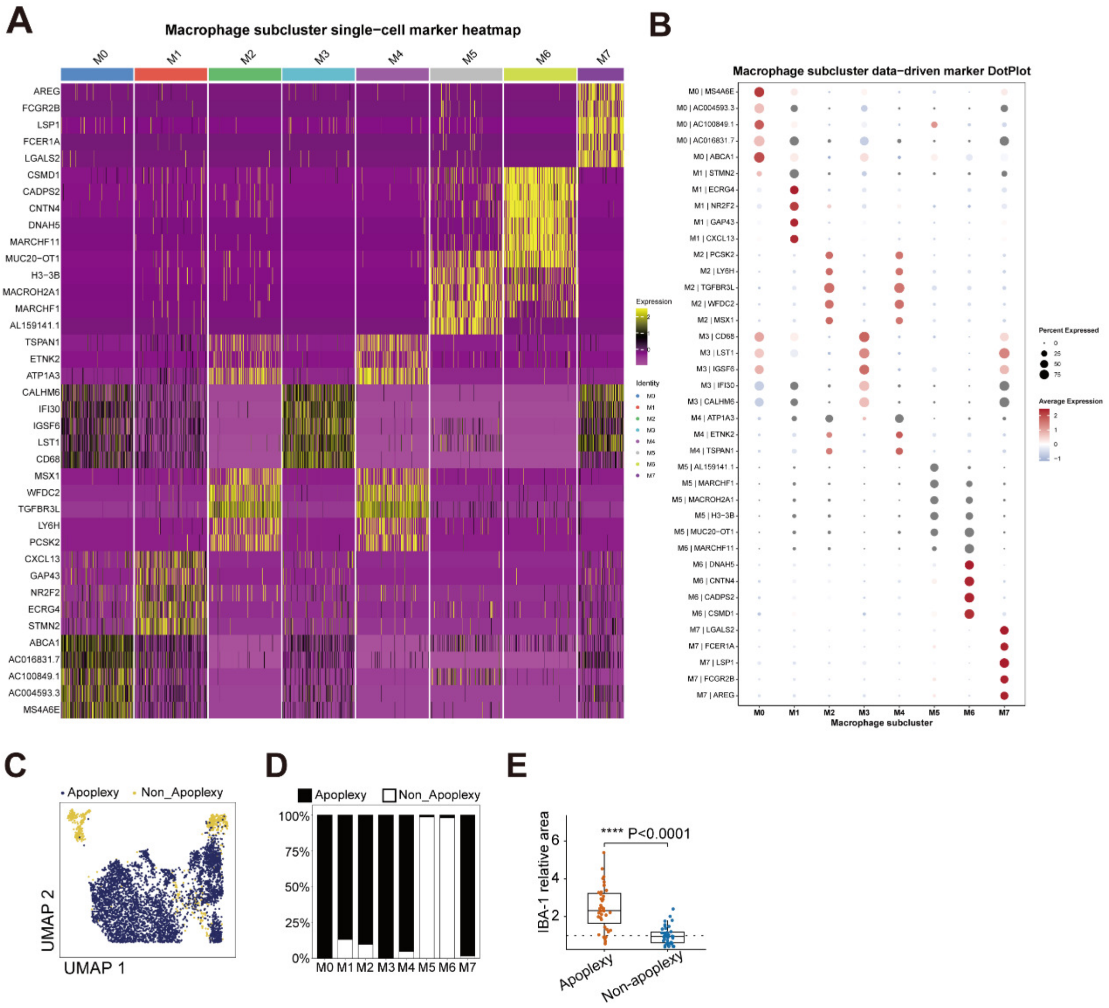

Supplementary Figure S2. Macrophage subcluster markers, group distribution, and additional tissue validation. (A) Marker-gene heatmap for the eight macrophage transcriptomic subclusters M0-M7 identified by unsupervised reclustering. (B) Dot plot showing representative marker genes used to characterize the macrophage subclusters. (C) UMAPs showing macrophage subcluster distribution in apoplectic and non-apoplectic samples. (D) Relative proportions of macrophage subclusters in the apoplexy and non-apoplexy groups. (E) Quantification of total IBA-1-positive relative area in apoplectic and non-apoplectic PitNET tissues, corresponding to the multiplex immunofluorescence analysis shown in the main figure. The M0-M7 labels indicate data-driven transcriptomic subclusters and do not denote predefined classical M0/M1/M2 macrophage states.

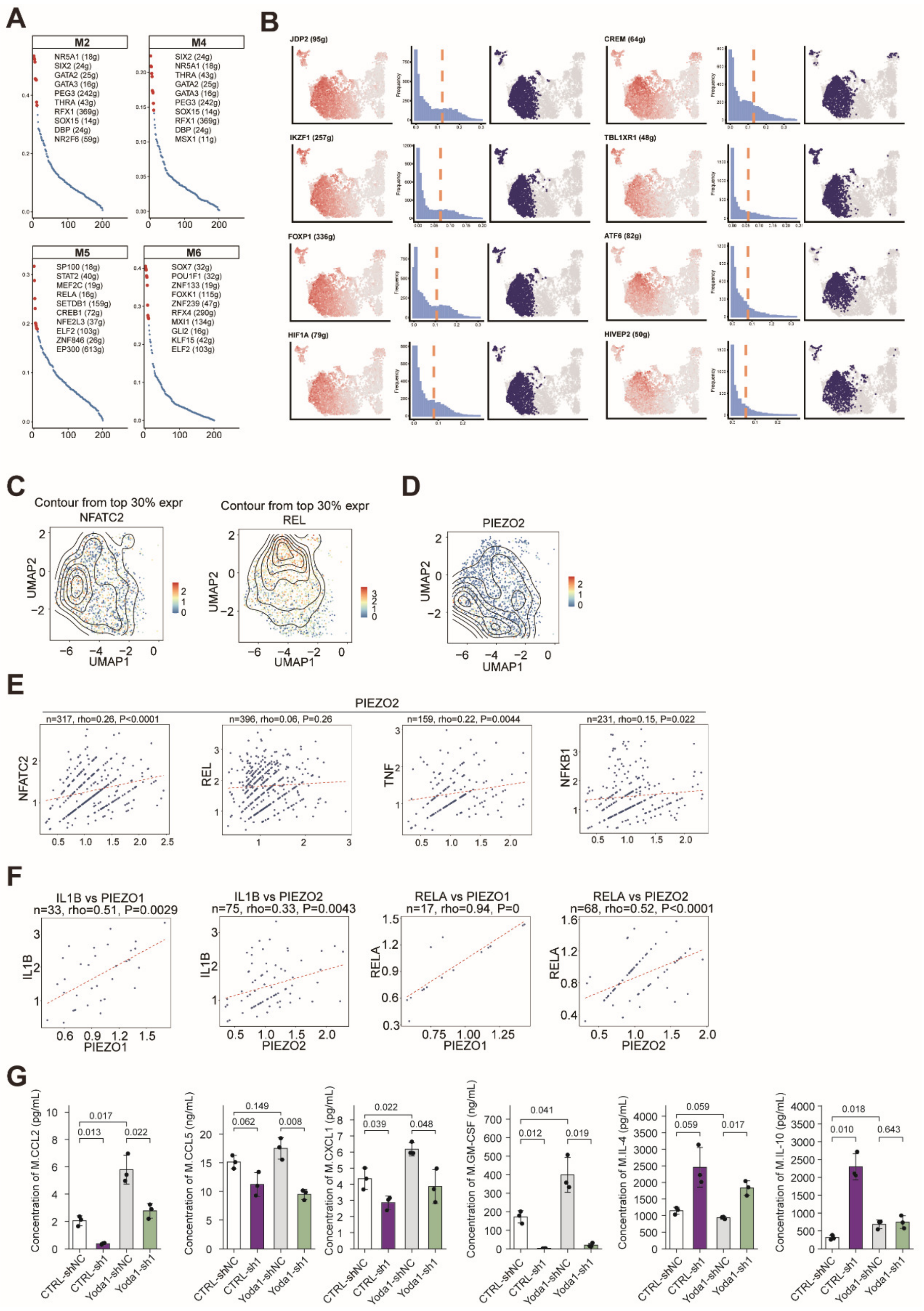

Supplementary Figure S3. Additional macrophage regulon, *PIEZO2*, and cytokine analyses. (A) Regulon specificity scores for additional macrophage subclusters not shown in the main figure. (B,C) Additional visualization of NFATC2 and REL regulon activity, including score distribution and active-cell mapping in macrophages. (D) *PIEZO2* expression in the macrophage UMAP. (E,F) Correlation analyses between *PIEZO2* and selected inflammatory

regulators or mediators, including *IL1B* and *RELA*. (G) Additional cytokine measurements in macrophage culture supernatants after Yoda1 stimulation, *Piezo1* knockdown, or related interventions. These supplementary analyses provide context for the PIEZO1-focused results shown in the main figure.

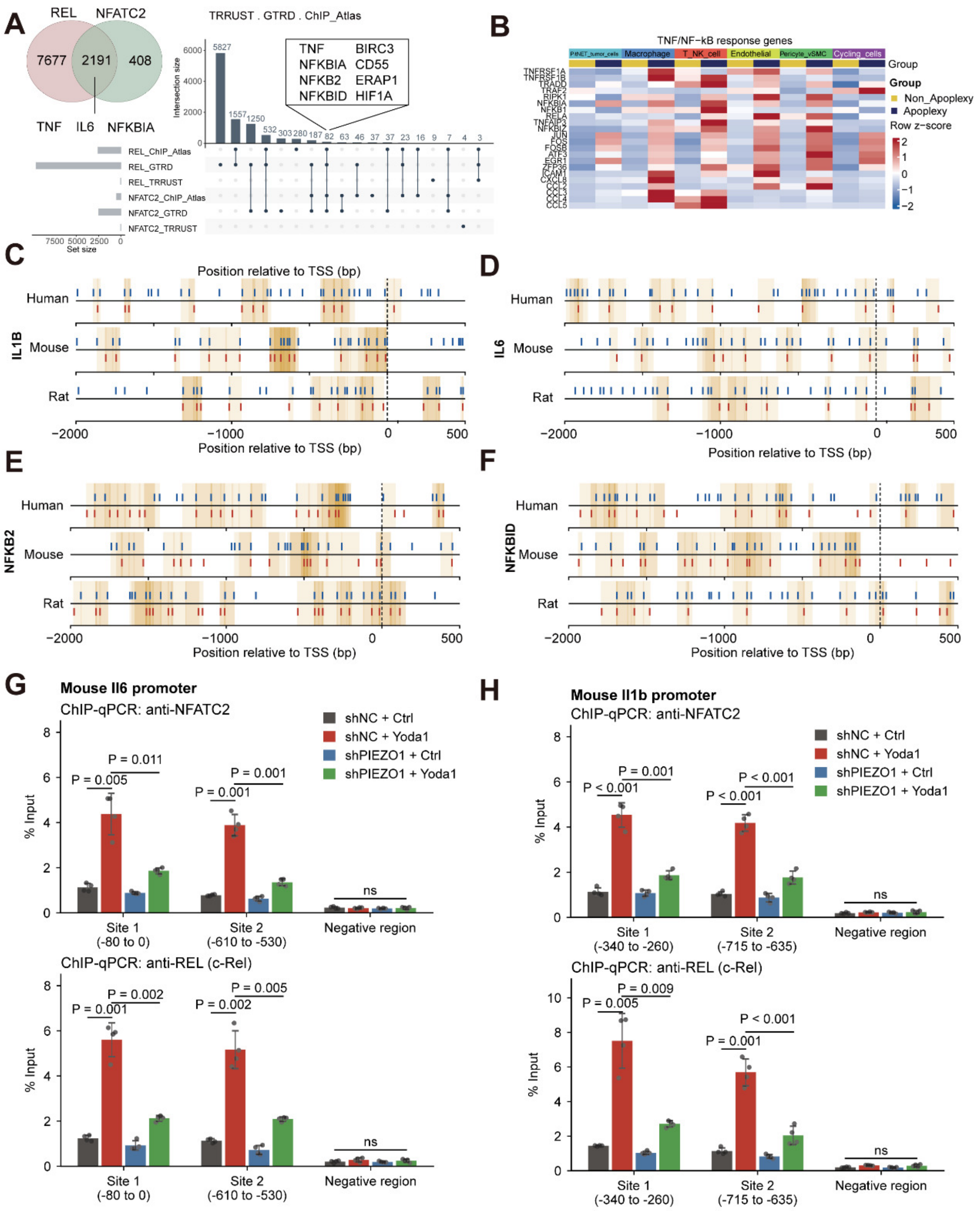

Supplementary Figure S4. Additional NFATC2/REL motif and ChIP-qPCR analyses. (A-F) Predicted NFATC2 and REL/c-Rel motif distributions in promoter regions of inflammation-related genes not fully shown in the main figure, including *IL1B*, *IL6*, *NFKB2*, and *NFKBID*, across human, mouse, and rat sequences where applicable. (G,H) ChIP-qPCR analysis of

NFATC2 and REL/c-Rel enrichment at *Il6* and *Il1b* promoter-associated sites in RAW264.7 macrophages after Yoda1 treatment with or without *Piezo1* knockdown. Negative-control regions were included where indicated. These analyses complement the *Tnf* promoter ChIP-qPCR results shown in the main figure.

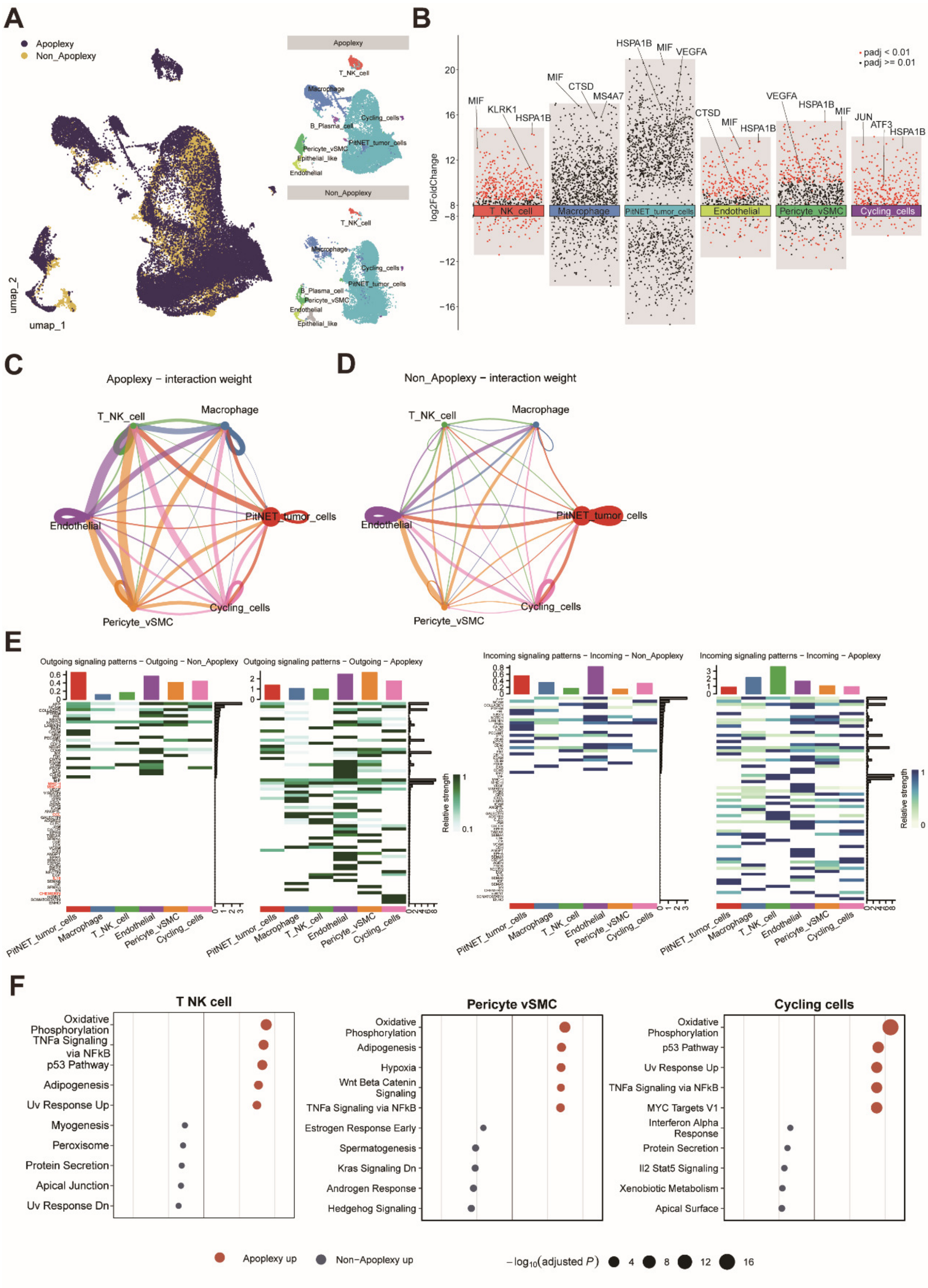

Supplementary Figure S5. Apoplexy-associated transcriptional changes, inferred

intercellular communication, and additional pathway enrichment. (A) Grouped UMAP visualization of major cell populations in apoplectic and non-apoplectic PitNET samples. (B) Differentially expressed genes or cell-type-specific transcriptional changes associated with the apoplexy group across major cell populations. (C,D) Additional CellChat-inferred communication networks and summary metrics comparing non-apoplectic and apoplectic samples. These CellChat summary metrics are presented descriptively. (E) Additional outgoing and incoming signaling patterns or relative information-flow analyses across ligand-receptor pathways. (F) Additional Hallmark pathway enrichment results for cell populations not fully shown in the main figure, including T and NK cells, pericytes/vSMCs, and proliferating/cycling cells.

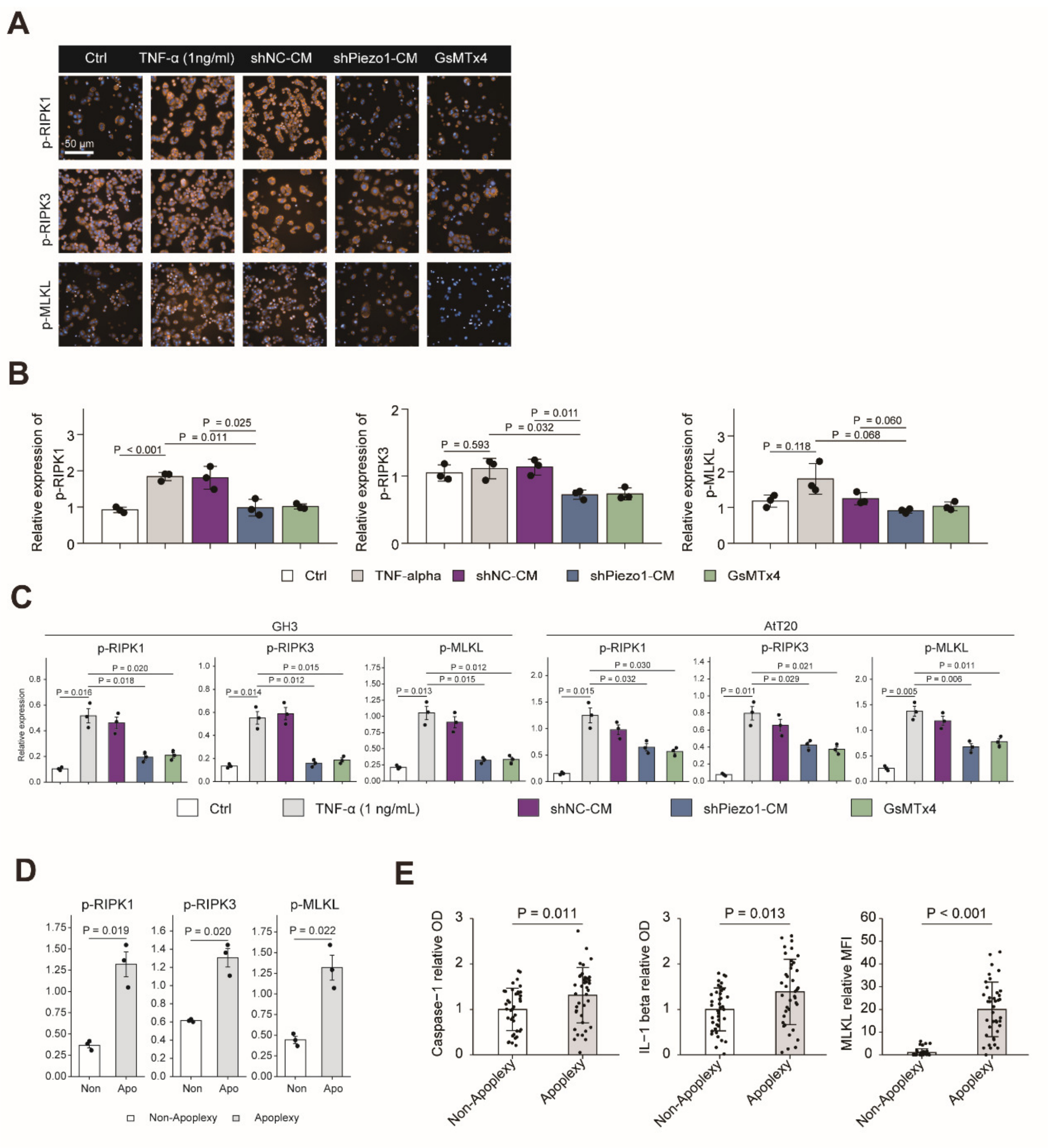

Supplementary Figure S6. Additional quantification of necroptosis-associated readouts in tumor cells, xenograft tissues, and clinical specimens. (A) Quantification of p-RIPK1, p-

RIPK3, and p-MLKL immunofluorescence signals in tumor cells after TNF-α-, conditioned-medium-, or inhibitor-related treatments. (B) Densitometric quantification of RIPK1/RIPK3/MLKL pathway Western blot readouts in GH3 and/or AtT-20 cells. (C) Quantification of p-RIPK1, p-RIPK3, and p-MLKL protein levels in tumor tissues from the shNC-RAW264.7 and shPiezo1-RAW264.7 xenograft groups. (D) Quantification of p-MLKL immunofluorescence signals in apoplectic and non-apoplectic clinical PitNET tissues. (E) Quantification of Caspase-1 and IL-1β immunohistochemical signals in apoplectic and non-apoplectic clinical PitNET tissues. These data provide supporting quantification for the necroptosis-associated analyses shown in the main figure.

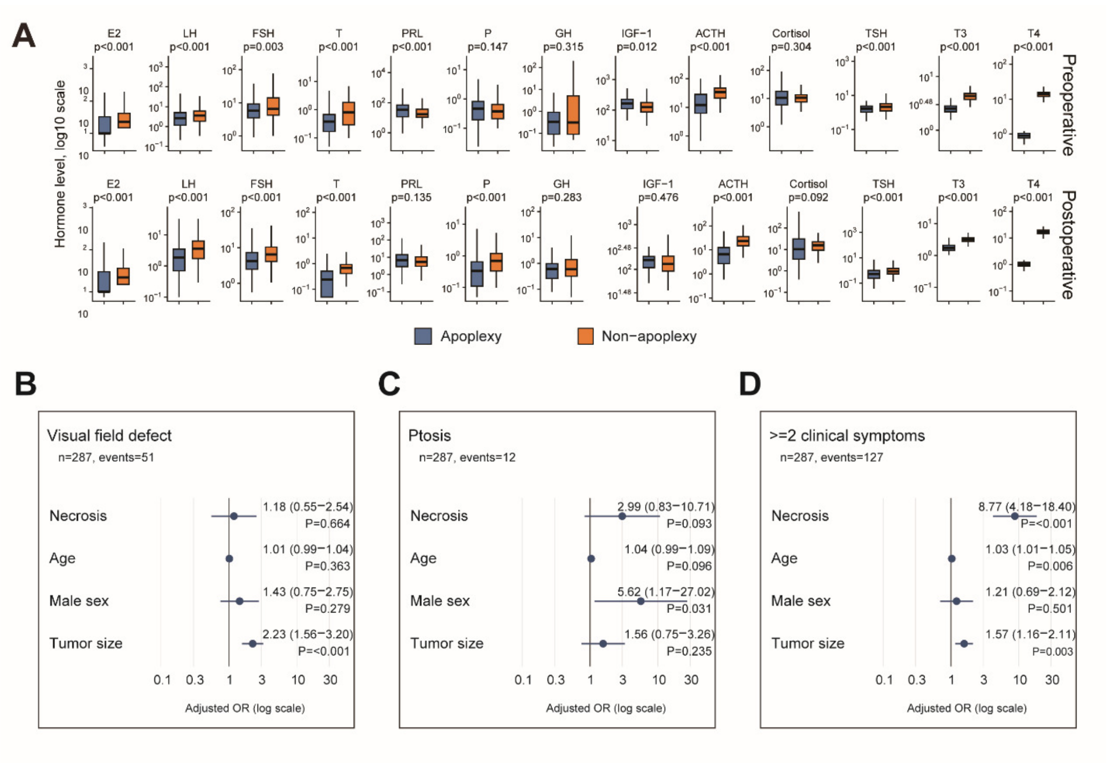

Supplementary Figure S7. Perioperative hormone profiles and additional clinical regression analyses in patients with pituitary apoplexy. (A) Preoperative and postoperative pituitary-related hormone profiles in the necrosis and non-necrosis subgroups, grouped by pituitary axes, including gonadal, thyroid, and ACTH-adrenal axes. (B-D) Additional multivariable logistic regression analyses evaluating the association between H&E-defined pathological necrosis and selected clinical outcomes after adjustment for age, sex, and tumor size. Results are presented as adjusted odds ratios with 95% confidence intervals for visual field defect, ptosis, and two or more clinical symptoms, respectively.

Supplementary Animal Experiments Attachment

ARRIVE 2.0-compliant description for in vivo pituitary tumor xenograft experiment

Animal experiments were designed to evaluate whether macrophage *Piezo1* knockdown altered necroptosis-associated signaling in pituitary tumor tissues. The in vivo model was established with reference to previous pituitary adenoma xenograft studies from the Department of Neurosurgery, Tongji Hospital, with modifications for macrophage intervention and necroptosis-associated molecular readouts. Six-week-old male BALB/c nude mice, weighing 18-22 g, were purchased from GemPharmatech. Male BALB/c nude mice were used because their immunodeficient background permits stable subcutaneous growth of pituitary tumor xenografts and because male mice allowed consistency within the animal cohort used in this experiment. The individual mouse was defined as the experimental unit.

Mice were housed under specific pathogen-free conditions at the Experimental Animal Center of Tongji Medical College, Huazhong University of Science and Technology. Animals were acclimatized for 7 days before experimentation and maintained in individually ventilated cages under controlled environmental conditions: temperature 22 +/- 2 °C, relative humidity 50 +/- 10%, and a 12-h light/12-h dark cycle. Sterile standard chow and autoclaved water were provided ad libitum. Bedding and nesting material were provided as environmental enrichment and were changed regularly according to institutional animal-care procedures.

The animal experiment used six mice in total, with three mice assigned to each group: the shNC-RAW264.7 group and the shPiezo1-RAW264.7 group (n=3 per group). This sample size was selected to provide independent biological replicates for comparing necroptosis-associated protein expression between groups while minimizing animal use according to the 3Rs principle. A formal a priori power calculation was not performed because the animal experiment was designed as a focused mechanistic validation experiment rather than as a tumor-growth, survival, or therapeutic-efficacy study.

For tumor establishment, equal numbers of GH3 cells were suspended in PBS (Servicebio)/Matrigel Matrix (Corning, 356234) at a 1:1 ratio and injected subcutaneously into the axillary region of each mouse. Each mouse received 1 x 10^7 GH3 cells in a final volume of 100 μL. On day 21 after tumor-cell implantation, mice bearing tumors of comparable volume were selected and randomly assigned to the shNC-RAW264.7 or shPiezo1-RAW264.7 group. shNC- or shPiezo1-treated RAW264.7 macrophages were then injected into the established tumors. Randomization was performed using computer-generated random numbers after matching tumors by volume. Tumor volume matching, treatment order, tissue-processing order, and Western blot loading order were balanced between groups whenever feasible to reduce potential confounding.

Animals were included if they were clinically healthy after acclimatization, successfully received the planned axillary tumor-cell implantation, and developed tumors suitable for macrophage intervention by day 21. Mice were excluded before randomization if tumor establishment failed, if tumors were not suitable for macrophage injection, or if severe illness unrelated to tumor implantation occurred. No

animals or data points were excluded from the final analysis after group allocation unless explicitly stated. Investigators responsible for endpoint tissue collection and Western blot quantification were blinded to group allocation by using coded sample labels until quantitative analysis was completed.

Animal health, body weight, tumor formation, tumor ulceration, mobility, grooming, feeding, drinking, and general behavior were monitored every 2 days after implantation and daily during the final week or whenever abnormal signs were observed. Humane endpoints included tumor ulceration or necrosis with skin breakdown, tumor diameter >15 mm, estimated tumor volume >1500 mm3, body-weight loss >20%, impaired mobility, severe lethargy, persistent hunching, poor grooming, reduced food or water intake, or any other sign of severe distress. No unexpected animal deaths or severe adverse events occurred before the planned endpoint.

The primary animal endpoints were tumor-tissue levels of necroptosis-associated proteins p-RIPK1, p-RIPK3, and p-MLKL measured by Western blot. According to the experimental timeline, tumor cells were implanted on day 0, macrophage intervention was performed on day 21 in tumors with comparable volume, and tumors were collected on day 28 for necrosis/necroptosis-associated assessment. At the experimental endpoint, mice were euthanized by CO2 inhalation followed by cervical dislocation. Tumor tissues were immediately harvested and processed for Western blot analysis of p-RIPK1, p-RIPK3, and p-MLKL, with beta-tubulin used as the loading control.

Supplementary Tables: Primers and Antibodies

Supplementary Table S1. Antibody information.

**Target**	**Supplier**	**Catalog**	**Dilution / amount**
IBA-1/Iba1	Abcam	ab178846	1:100
CD4	Abcam	ab133616	1:500
CD8 alpha	Abcam	ab237709	1:2000
CD19	Abcam	ab320735	1:2000
HLA-DR	Abcam	ab92511	IF 1:100
CD206	Abcam	ab64693	IF 1:100
PIEZO1/Piezo1	Abcam	ab128245	IHC 1:100; IF 1:100
TNF-α	Abcam	ab225576	1:100–1:200
p-RIPK1	Proteintech	28252-1-AP	WB1:4000;
p-RIPK3	Proteintech	87148-1-RR	WB 1:4000;
p-MLKL	Proteintech	82090-2-RR	IHC-P 1:250; IF 1:100; WB 1:5000
Caspase-1	Abcam	ab138483	IHC-P 1:50; IF 1:100
IL-1β	Abcam	ab315084	IHC-P 1:500; IF 1:50
NFATC2	Santa Cruz Biotechnology	sc-7296 X	5 μg/IP
REL/c-Rel	Santa Cruz Biotechnology	sc-6955 X	5 μg/IP
normal IgG	Santa Cruz Biotechnology	sc-2025	5 μg/IP
NFATC2	ImmunoWay	YM9023	1:10000
REL/c-Rel	ImmunoWay	YM9393	1:1000
β-tubulin	Proteintech	66240-1-Ig	1:100000
Lamin B1	Proteintech	12987-1-AP	1:6000

Supplementary Table S2. shRNA target sequences used in this study.

**shRNA**	**Target sequence (5'-3')**	**Description**
hNC	TTCTCCGAACGTGTCACGT	Non-targeting negative control
shPiezo1-1	CGATGATGACGACGATGAAGA	*Piezo1* knockdown
shPiezo1-2	GGCTATTTGGTCTCAAGAACT	*Piezo1* knockdown
shPiezo1-3	CCTTGTTCCAGGTCTACTACA	*Piezo1* knockdown

Note: hNC indicates the non-targeting negative-control shRNA. All sequences are listed in the sense orientation from 5' to 3'.

Supplementary Table S3. Primer information.

Assay	Gene / amplicon	Forward primer (5′–3′)	Reverse primer (5′–3′)	Region / note
RT-qPCR	*Piezo1*	CTTACACGGTTGCTGGTTGG	CACTTGATGAGGGCGGAAT	Mouse
RT-qPCR	*Nos2*/iNOS	GACATTACGACCCCTCCCAC	GCACATGCAAGGAAGGGAAC	Mouse
RT-qPCR	*Tnf*	AGACCCTCACACTCAGATCATCTTC	CCACTTGGTGGTTTGCTACGA	Mouse
RT-qPCR	*Il1b*	GCCACCTTTTGACAGTGATGAG	GTTTGGAAGCAGCCCTTCATC	Mouse
RT-qPCR	*Il6*	CAACGATGATGCACTTGCAGA	TCTCTCTGAAGGACTCTGGCT	Mouse
RT-qPCR	*Gapdh*	CATCACTGCCACCCAGAAGACTG	ATGCCAGTGAGCTTCCCGTTCAG	Mouse internal control
ChIP-qPCR	*Tnf* Site 1	GGACCTCACAAGCCTTCTCC	AGAAATCCTGGAAGCAGGGC	chr17:35421643–35421801; 159 bp
ChIP-qPCR	*Tnf* Site 2	GTAGAAAGACCATGCCTGTGTC	TCAGCCACTTCCTCCAAGAAC	chr17:35421359–35421524; 166 bp
ChIP-qPCR	*Tnf* Negative control	CACTGTCCTTCTTGCCCTCC	GGTGCCTATGTCTCAGCCTC	chr17:35420563–35420731; 169 bp
ChIP-qPCR	*Il6* Site 1	AGGTTTCCAATCAGCCCCAC	GTTCTTGGTGGGCTCCAGAG	chr5:30218000–30218166; 167 bp
ChIP-qPCR	*Il6* Site 2	AGACCTTCAAGCCTCCTTGC	CTCTGCATTCTCCCCAGTGG	chr5:30217233–30217381; 149 bp
ChI	*Il6*	CACGGCCTTCCCTACT	AGCCTCCGACTTGT	chr5:3021

P-qPCR	Negative control	TCAC	GAAGTG	8420–30218514; 95 bp
ChIP-qPCR	*Il1b* Site 1	GTCTGATAATGCCAGGGTGC	AGGAAAGCAGGAGTGGGTG	chr2:129213646–129213815; 170 bp
ChIP-qPCR	*Il1b* Site 2	TTCGCAAGTGTGTCATCGTG	TGTGCATCTACGTGCCTACC	chr2:129213271–129213422; 152 bp
ChIP-qPCR	*Il1b* Negative control	CCCAAAGTCCATCAGTGGGG	TGTCTCTGTCTCTCTCTGTCTG	chr2:129212749–129212858; 110 bp
